# Regio- and Stereoregular EVOH Copolymers from ROMP as Designer Barrier Materials

**DOI:** 10.1021/acspolymersau.4c00006

**Published:** 2024-04-11

**Authors:** Claire E. Dingwell, Marc A. Hillmyer

**Affiliations:** Department of Chemistry, University of Minnesota, Minneapolis, Minnesota 55455, United States

**Keywords:** functional polyolefins, gas barrier materials, metathesis, semicrystalline, packaging

## Abstract

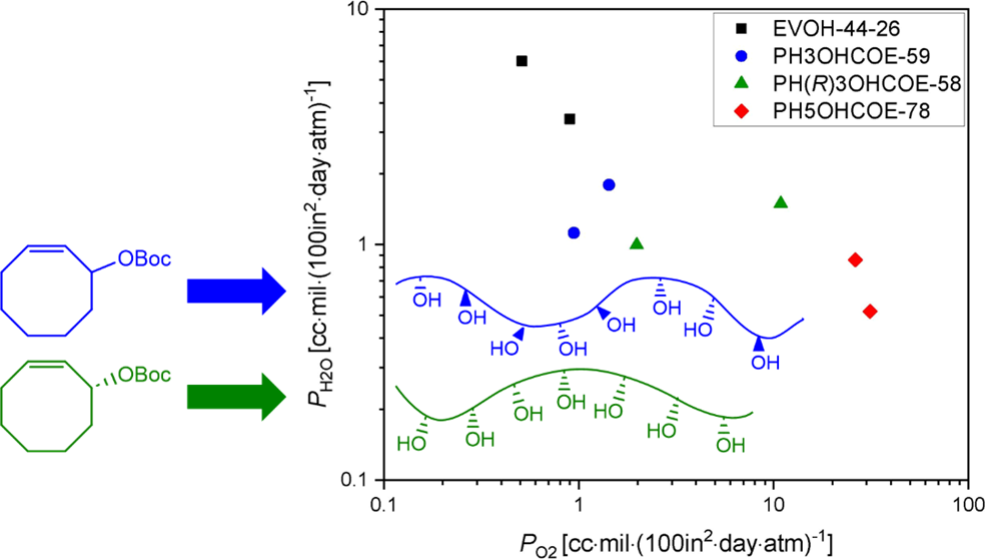

This work aimed to decrease the water permeability (*P*_H2O_) while simultaneously maintaining low oxygen permeability (*P*_O2_) in ethylene vinyl alcohol (EVOH)-based copolymers by introducing high levels of backbone regioregularity and stereoregularity. Both regioregular atactic and isotactic EVOH samples with 75 mol % ethylene were prepared by a ring-opening metathesis polymerization (ROMP)-hydrogenation-deprotection approach and then compared to commercial EVOH(44) (containing 44 mol % ethylene) as a low *P*_O2_ standard with poor water barrier characteristics (i.e., high *P*_H2O_). The high levels of regioregularity and stereoregularity in these copolymers increased the melting temperature (*T*_m_), degree of crystallinity (χ_c_), and glass-transition temperature (*T*_g_) compared to less regular structures. EVOH(44) demonstrated the highest *T*_m_ but lower χ_c_ and *T*_g_ values as compared to that of the isotactic polymer. Wide-angle X-ray scattering showed that semicrystalline EVOH(44) exhibited a monoclinic structure characteristic of commercial materials, while ROMP-derived polymers displayed an intermediate structure between monoclinic and orthorhombic. Tensile testing showed that isotacticity resulted in brittle mechanical behavior, while the atactic and commercial EVOH(44) samples had higher tensile toughness values. Although EVOH(44) had the lowest *P*_O2_ of the samples explored, the atactic and tough ROMP-derived polymer approached this value of *P*_O2_ while having a *P*_H2O_ over 3 times lower than that of commercial EVOH(44).

Ethylene vinyl alcohol (EVOH) is a commercial copolymer synthesized by free-radical copolymerization of ethylene and vinyl acetate, followed by deacylation.^1^ It is used primarily as a barrier packaging material that prevents premature oxygen-induced degradation of food and other goods.^2^ The only commercially relevant polymer with lower oxygen permeability (*P*_O2_) than EVOH is poly(vinyl alcohol) (PVOH), but PVOH is difficult to process due to low degradation temperatures (*T*_d_) and high affinity for water. As expected, the incorporation of ethylene units along the backbone leads to better water resistance, higher *T*_d_ values, and processability at the cost of increased *P*_O2_ values.^3^ However, at copolymer compositions necessary for practical oxygen barrier materials (24–48 mol % ethylene),^4^ outer polyolefin layers are typically required to provide the necessary low *P*_H2O_ values given the high water permeability of EVOH alone.^5^

Gas transport across a membrane requires a gas molecule to dissolve in a material, diffuse through it, and desorb on the opposite side. Nonpolar gas molecules, such as oxygen, are not very soluble in polar materials, such as EVOH; polar molecules, such as water, are highly soluble. Once a gas molecule is dissolved in the material, increased crystallinity and decreased free-volume primarily decrease its diffusion rate.^6−8^ Gas molecules diffuse through amorphous regions of a semicrystalline material, so increasing the degree of crystallinity (χ_c_) leads to decreases in permeability.^9,10^ Increasing tortuosity in the amorphous regions also hinders diffusion of dissolved molecules.^8^ This is accomplished by decreasing free-volume through increased level and strength of intermolecular interactions, typically with concomitant increases in the glass-transition temperature (*T*_g_) of the material.^8,11,12^

Increasing the structural regularity of semicrystalline polymers through decreases in branching or increases in regio- and stereoregularity often leads to increases in χ_c_,^13^ intermolecular interactions, and *T*_g_;^14^ however, free-radical polymerization offers little control over structural regularity in EVOH synthesis.^15^ We recently probed the effect of regioregularity on the barrier properties of EVOH with high (75 mol %) ethylene content derived from ring-opening metathesis polymerization (ROMP) in an attempt to simultaneously achieve very low values of *P*_O2_ by virtue of regular placement of hydroxyl groups and *P*_H2O_ from the high ethylene content, targeting a monomaterial with both practically low oxygen and water permeability.^16^ Those materials were good water barrier materials, and regioregular polymers gave decreased *P*_O2_ values compared to those of regioirregular samples. However, these polymers did not achieve the ultralow *P*_O2_ values (high oxygen barrier characteristics) of the benchmark commercial EVOH. So, we turned our attention to including both regio- and stereoregularity in the polymers.

The preparation of stereoregular polymers by ROMP is more challenging due to the difficult monomer synthesis required. Scherman et al.^17^ polymerized *cis*- and *trans*-5,6-acetonidecyclooctene, followed by hydrogenation and deprotection, to give an EVOH-based polymer with controlled stereochemistry in each individual repeat unit. Due to the lack of regioselectivity, however, the polymers would not be described as stereoregular (e.g., isotactic). Choi and Hong have recently reported the polymerization of similar strained monomers based on cyclohexene instead of cyclooctene, along with routes toward chemical recycling of these polymers containing higher concentrations of vinyl alcohol.^18^ Recent work has utilized regioselective ROMP of (*R*)- or (*S*)-3-substituted cyclooctene and cyclopentene to achieve isotacticity.^19−21^ Zhang et al.^21^ synthesized isotactic ethylene vinyl acetate from regiospecific ROMP-hydrogenation of 3-acetoxycyclooctene (3AcCOE), but deprotection was not performed. Recently, Guillory et al.^22^ synthesized and polymerized (*R*)- and (*S*)-3-(*tert*-butyl-dimethylsiloxy)cyclopentene, followed by hydrogenation and deprotection, to give regioregular, isotactic EVOH with hydroxyl groups located every five carbons along the backbone (60 mol % ethylene content); however, the barrier properties of these materials were not examined. Further work was recently reported by Tashiro et al.^23^ on the detailed analysis of the crystal structure of this polymer. In this work, we investigate the barrier properties of a regioregular, isotactic EVOH polymer with 75 mol % ethylene to compare the barrier performance with atactic and regiorandom derivatives with the hypothesis that even higher degrees of order in the polymeric backbone will lead to enhanced intermolecular interactions, higher levels of crystallinity, higher glass-transition temperatures, and even higher water and oxygen barrier properties in a single material.

We prepared racemic 3-*tert*-butoxycarbonate-cyclooctene (3OBocCOE) via protection of 3-hydroxycyclooctene^21^ with Boc anhydride. Palladium-catalyzed substitution of (*S*)3OBocCOE with sodium benzenesulfinate by Pd_2_(dba)_3_·CHCl_3_ and the (*R*,*R*)-DACH-phenyl Trost ligand allowed for the isolation of (*R*)3OBocCOE^21,24^ with >99% *ee* by Mosher ester analysis (Figures S.5–S.9).^25^ Using the Grubbs Second Generation catalyst (G2) in toluene-*d*_8_, the polymerization of 3700 equiv of 3OBocCOE was complete after 70 min, with first-order dependence on 3OBocCOE (Figures S.10–S.12). Interestingly, this polymerization occurred faster than that of 3AcCOE, despite the steric bulk of the *tert*-butyl group (Figure S.13). This could be due to the increased distance between the bulky alkyl group and the catalyst by virtue of the extra oxygen atom in the carbonate as compared with the acetate.

3OBocCOE and (*R*)3OBocCOE were successfully polymerized by G2 in the presence of *cis*-4-octene as a chain-transfer agent with full conversion and high isolated yields to give polyalkenamers P3OBocCOE and P(*R*)3OBocCOE (Scheme 1, Table S.1). Both samples had high regioregularity (>99% HT) and >90% *E* stereochemistry, as expected.^19,20^ After hydrogenation and deprotection (Scheme 1), PMMA-relative molar mass values were measured by size-exclusion chromatography (SEC) in hexafluoroisopropanol (HFIP) (Table S.2). Additionally, we compared the samples synthesized in this study to a commercial EVOH(44) polymer (44 mol % ethylene) made by free-radical copolymerization as well as a regiorandom polymer made by ROMP-hydrogenation-deprotection of 5AcCOE as reported in our previous study.^16^ The structures, names, and molar masses in kg·mol^–1^ of these polymers are provided in Scheme 1.

**Scheme 1:**
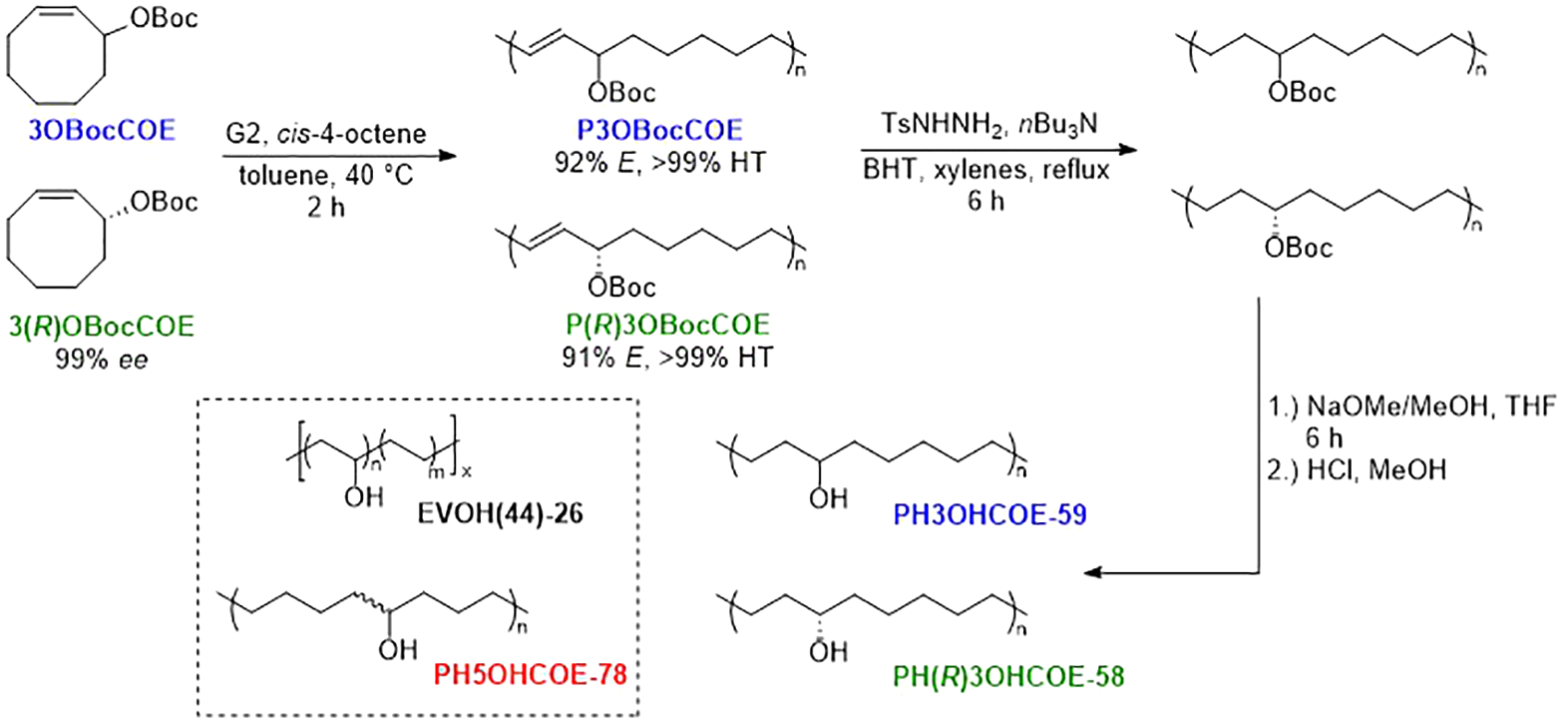
Synthesis of Atactic and Isotactic EVOH from ROMP of 3OBocCOE and (R)3OBocCOE Relative molar mass of final polymers (HFIP-SEC against PMMA Standards) in kg·mol^–1^ is shown by the number at the end of the polymer name. EVOH(44)-26 is a commercial sample, and PH5OHCOE-78 was prepared in a previous study.^16^

Although the isotactic polymer was soluble at low concentrations in HFIP and TFA-*d* for SEC and NMR analysis, respectively, we were unable to achieve reasonable concentrations necessary for drop-casting (typically >10 mg·mL^–1^). As such, we turned to melt-processing methods. Cerrada et al.^26^ have demonstrated that melt-processing EVOH copolymers containing 29, 32, and 44 mol % ethylene with a low cooling rate can result in a monoclinic crystal structure, leading to higher levels of hydrogen-bonding between chains. We melt-pressed powdered samples of each polymer at 15 000 psig (103 MPa) for 3 min, 15 °C above their respective *T*_m_ values, then cooled the samples in the press at a rate of ∼25 °C·min^–1^ to room temperature, resulting in films with thicknesses around 100 μm that were then annealed at 115 °C for 2 h under inert atmosphere, except PH5OHCOE, which was annealed at 85–90 °C for 2 h due to its lower melting temperature (*T*_m_ = 120 °C). All films exhibited high *T*_d,5%_ values that were far above their respective melting *T*_m_ values by thermogravimetric analysis (TGA) and differential scanning calorimetry (DSC) (Table 1, Figure 1), respectively. DSC of the films post processing (Table 1) showed that regiorandom PH5OHCOE-78 had the lowest *T*_g_, *T*_m_, enthalpy of melting (Δ*H*_m_), and, as a result, lowest χ_c_. Regioregular, atactic PH3OHCOE-59 had higher values of *T*_g_, *T*_m_, Δ*H*_m_, and χ_c_ in comparison to the regiorandom sample. Isotacticity in the PH(*R*)3OHCOE-58 sample led to even further increases in these values. Although commercial EVOH(44)-26 had a high *T*_g_ comparable to isotactic polymers and the highest *T*_m_ of all samples due to its lower ethylene content, it exhibited χ_c_ values between those of PH3OHCOE-59 and PH(*R*)3OHCOE-58. Values of *T*_c_ and Δ*H*_c_ trended well with the values from the corresponding melting transitions. While PH3OHCOE-59 had the lowest density (ρ), followed by PH(*R*)3OHCOE-58, regiorandom PH5OHCOE-78 had the second highest ρ, which was unexpected due to its lower χ_c_ and lack of structural regularity. Only EVOH(44)-26 had a higher ρ, which is again attributed to higher vinyl alcohol content (56 mol % vinyl alcohol).

**Table 1:** Thermal and Mechanical Properties of ROMP-Derived and Commercial EVOH Polymers^a^

	*T*_d_ (°C)^b^	*T*_g_ (°C)^c^	*T*_m_ (°C)^c^	Δ*H*_m_ (J·g^–1^)^c^	χ_c_^d^	*T*_c_ (°C)^e^	Δ*H*_c_ (J·g^–1^)^e^
PH5OHCOE-78	419	83	120	39	0.16	100	42
PH3OHCOE-59	431	88	140	58	0.23	121	64
PH(*R*)3OHCOE-58	439	106	174	92	0.37	157	98
EVOH(44)-26	331	97	188	72	0.35	167	65

	ρ (g·mL^–1^)^f^	*E* (GPa)^g^	ε_b_ (%)^g^	σ_b_ (MPa)^g^	Toughness^g^
PH5OHCOE-78	1.18	0.63^h^	260^h^	4.6^h^	33^h^
PH3OHCOE-59	1.07	0.94	37	58	17
PH(*R*)3OHCOE-58	1.11	1.0	3.2	19	0.54
EVOH(44)-26	1.23	1.8	12	41	4.8

a Data was collected on melt-pressed and annealed samples unless specified by footnote h.

b Data obtained from TGA (10 °C·min^–1^, N_2_ atmosphere, 5% mass loss).

c Data obtained from DSC (10 °C·min^–1^, first heating cycle).

d Calculated with Δ*H*_m_ [χ_c_ = (Δ*H*_m_/Δ*H*_f(100)_)·100].^27^ Δ*H*_f(100)_ (enthalpy of fusion for a 100% crystalline polymer) was calculated for each polymer type based on weight fraction (*w*) and literature Δ*H*_f(100)_ values [Δ*H*_f,100_ = *w*_PE_(Δ*H*_f(100), PE_) + *w*_PVOH_(Δ*H*_f(100), PVOH_)].^28^

e Data obtained from DSC (10 °C·min^–1^, cooling cycle).

f Determined by Archimedes method using a Mettler-Toledo scale.

g Data obtained from a Shimadzu AGS-X (1 mm·min^–1^), reported as an average of five replicates per sample type, excluding outliers.

h Data recorded from previous study of films drop-cast from HFIP, followed by slow evaporation and annealing at 85 °C for 2 h.

**Figure 1:**
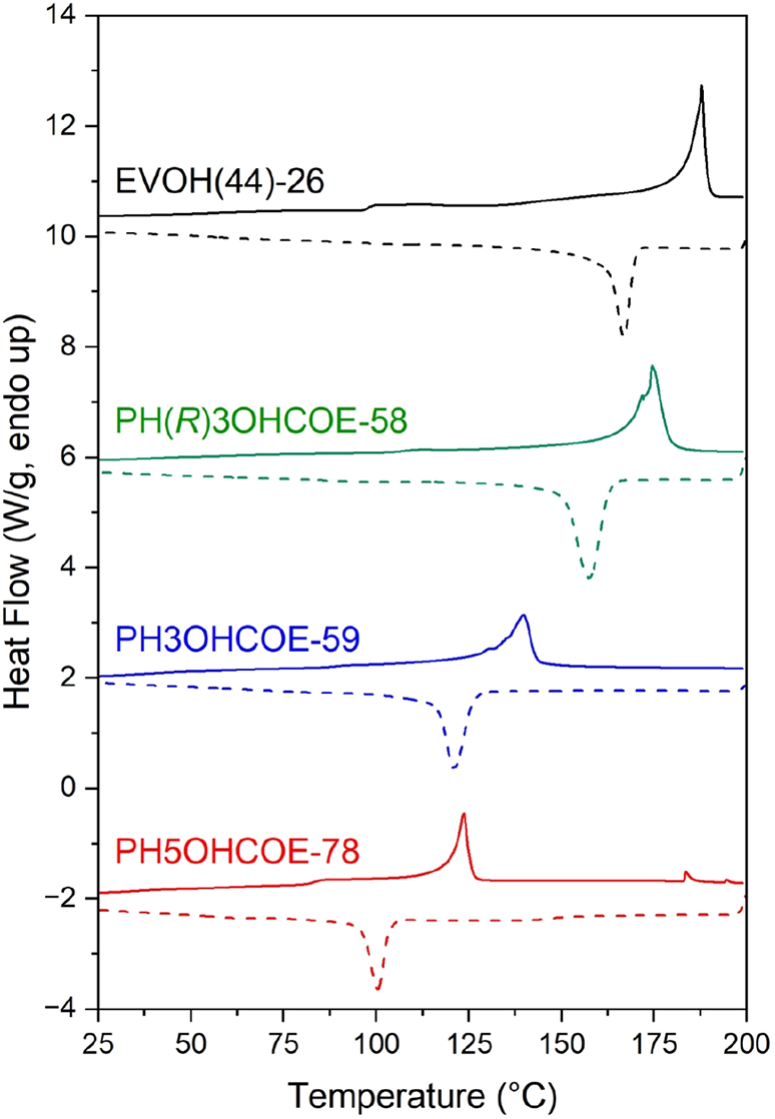
Heating (solid lines) and cooling (dashed lines) cycles from DSC (10 °C·min^–1^) of melt-pressed polymers. Traces were shifted vertically for clarity.

The crystal structure of EVOH depends on the copolymer composition, with ethylene-rich polymers exhibiting an orthorhombic crystal structure, vinyl alcohol-rich polymers exhibiting a monoclinic crystal structure induced by intermolecular hydrogen-bonding, and the intermediate region exhibiting an intermediate or “pseudohexagonal” crystal structure between the two crystal types, which is composition- and processing-dependent.^5,26,29,30^ We were unable to determine the crystal structure of our ROMP-derived polymers from nonoriented samples, so the WAXS patterns after comparable processing histories were compared to literature EVOH scattering patterns (Figure 2). The EVOH(44)-26 sample exhibited a monoclinic crystal structure when compared to a polymer with a similar copolymer composition (Figure S.43).^30^ However, the ROMP-derived polymers appeared to be exhibiting the intermediate “pseudohexagonal” structure based on the broadened appearance of the lowest *q* reflections between 1.20–1.65 Å^–1^ (Figure S.42, 8–11° on 2θ axis), caused by the merging of [101̅], [101], and [200] reflections of the monoclinic crystal structure as the ethylene content increases.^30^ PH3OHCOE-59 and PH(*R*)3OHCOE-58 had a low-angle peak from the [100] plane of the monoclinic crystal structure, indicating that the polymers had some monoclinic character. Unlike the isotactic polymer, PH3OHCOE-59 had very sharp peaks near 2.25 and 3.29° Å^–1^ on the *q* axis that initially appeared to be from impurities (Figure S.44). Another sample of this material was analyzed to rule out impurities; some peaks were still present but less pronounced and broadened. When compared to literature peaks from PVOH, these aligned well with the [002] and [401̅] reflections from the monoclinic crystal structure; see Figures S.45–S.49. Comparatively, the isotactic polymer had numerous peaks located beyond 1.65 Å^–1^ that could align with monoclinic or orthorhombic structures, again pointing to a more intermediate structure (Figures S.50–S.54). Previous work on isotactic PVOH has shown that intermolecular hydrogen-bonding is reduced with increasing isotacticity, but the polymers in our study are rich in ethylene, making direct comparisons to this work challenging with increased distance between hydroxyl groups along the chain.^31^ WAXS of PH5OHCOE-78 has been previously reported and appears to adopt the discussed intermediate crystal structure.^16^

**Figure 2:**
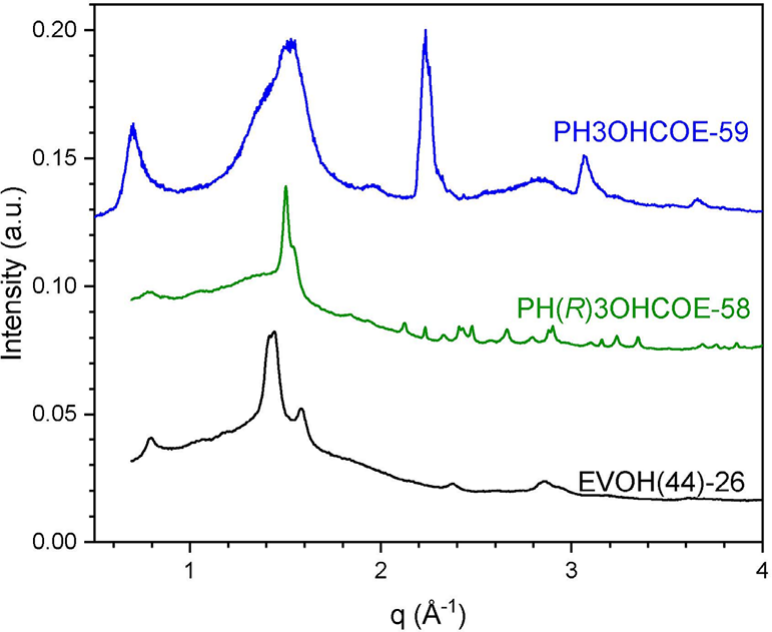
1D WAXS of melt-pressed ROMP-derived and commercial EVOH. Traces are shifted vertically for clarity. PH(*R*)OHCOE-58 and EVOH(44)-26 were analyzed at Argonne National Lab (λ = 0.729 Å), and PH3OHOE-59 was analyzed at UMN with a Mo source (λ = 0.711 Å).

Two thermal transitions, *T*_g_ and *T*_m_, are evident by DMTA upon the precipitous decreases in storage modulus (*E*′) (Figure S.55). *T*_g_ is noted by the maximum value of tan(δ) near the first *E*′ drop (Figure S.56), and while the values from DMTA roughly correlated to those from DSC, they were lower and in the range of 44–54 °C (Table S.3). *T*_m_ values were very similar to those from DSC, but only EVOH(44)-26 exhibited the expected crossover of *E*′ and the loss modulus (*E*″). The atactic and isotactic 3-substituted samples displayed a drop in both moduli, but no crossover, indicative of a lack of terminal flow. Although this could be due to cross-linking, this result was observed in our previous study and was attributed to a potential hydrogen-bonding effect made possible by linear chains with regular spacing between −OH groups.^16^ This is supported by POM (see POM video of EVOH(44)-26, POM video of PH3OHCOE-59, and POM video of PH(R)3OHCOE-58 in Supporting Information), where birefringence is observed above *T*_m_.

The processing protocol used in this work (melt-press followed by cooling in press) led to different tensile properties of EVOH(44)-26 and PH3OHCOE-59 compared to our previous reports on drop-cast films, with samples exhibiting higher values of Young’s Modulus (*E*), toughness, strain-at-break (ε_b_), and stress-at-break (σ_b_) (Table 1, Figure S.57).^16^ In fact, some trials of EVOH(44)-26 exhibit yield point and ductile behavior. PH(*R*)3OHCOE-58 had lower values of toughness, ε_b_, and σ_b_ compared to the atactic and commercial samples; however, this sample exhibited moderate values of *E* between PH3OHCOE-59 and EVOH(44)-26. *E* trended well with χ_c_ as expected. Due to material limitations, we did not measure the tensile properties of melt-pressed PH5OHCOE-78 and report the tensile data from our previous study of films drop-cast from HFIP, followed by slow evaporation and annealing at 85 °C for 2 h in Table 1.^16^

*P*_O2_ and *P*_H2O_ values were determined from the oxygen and water transmission rates (Figure 3, Table S.4). EVOH(44)-26 still exhibited the lowest *P*_O2_ [0.71 cc·mil·(100 in^2^·day·atm)^−1^] . However, the *P*_O2_ of PH3OHCOE-59 [1.2 cc·mil·(100 in^2^·day·atm)^−1^] was only about 50% higher than EVOH(44)-26 using these processing conditions, and one sample achieved 0.9 cc·mil·(100 in^2^·day·atm)^−1^. These values were significantly lower than in our previous study on drop-cast films that were quickly pressed to remove pinholes, despite PH3OHCOE-59 having lower values of *T*_g_, χ_c_, and ρ than the corresponding PH3OHCOE-215 sample.^16^ We hypothesize that cooling slowly in the melt-press allowed more intermolecular hydrogen-bonds to form before chain mobility is decreased below the *T*_c_ and *T*_g_. This is supported by DMTA and POM, where the lack of moduli crossover and birefringence above *T*_m_ are present below the molar mass threshold where these effects were observed previously. Additionally, the crystal structure showed more peaks potentially from the monoclinic structure, supporting the idea of increased hydrogen-bonding.^29,32^ Interestingly, PH(*R*)3OHCOE-58 had a higher average value of *P*_O2_ [6.4 cc·mil·(100 in^2^·day·atm)^−1^], although close to that of PH3OHCOE-59, despite having higher values of *T*_g_, χ_c_, and ρ. Higher values of χ_c_ are not always indicative of low *P*_O2_; for example, high-density polyethylene has a very high χ_c_ but high *P*_O2_.^7,33^ Previous literature comparing isotactic and atactic PVOH showed that isotactic polymers likely have lower χ_c_ and lower intermolecular hydrogen-bonding due to the higher ability of intramolecular hydrogen-bonding between adjacent hydroxyl groups; however, the spacing between hydroxyl groups along the backbone in these EVOH samples would not allow for intramolecular hydrogen-bonding between adjacent groups.^34,35^ This result coupled with the past literature suggests that the more complex monomer synthesis required to make isotactic polymers is not necessary to achieve low values of *P*_O2_. PH5OHCOE-78 that has been melt-pressed had the highest *P*_O2_ with the lowest χ_c_ and *T*_g_.

**Figure 3:**
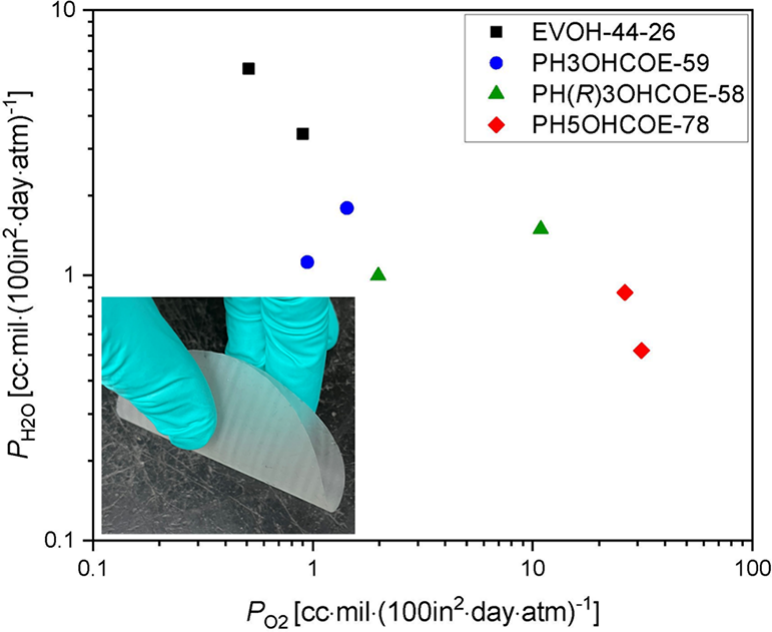
*P*_O2_ vs *P*_H2O_ for EVOH(44)-26 (black square), PH3OHCOE-59 (blue circle), PH(*R*)OHCOE-58 (green triangle), and PH5OHCOE-78 (red diamond) processed by melt-pressing for 3 min, then cooling to room-temperature in press. Photo of representative film (PH5OHCOE-78) shown to demonstrate flexibility. Two trials were tested per sample type; each sample type has two points representing these two trials.

Finally, *P*_H2O_ was quite similar for PH3OHCOE-59 and PH(*R*)3OHCOE-58. A slightly lower *P*_H2O_ was observed for PH5OHCOE-78, which was not observed in our previous study on drop-cast films.^16^ However, EVOH(44)-26 had a *P*_H2O_ over 3 times higher due to its higher vinyl alcohol content. Remarkably, with this melt-processing method, PH3OHCOE-59 can achieve *P*_O2_ values nearly as low as EVOH(44)-26 while maintaining lower *P*_H2O_ than EVOH(44)-26, opening up possibilities for a monomaterial with both high water and oxygen barrier properties for a next-generation package through macromolecular design.

In conclusion, atactic and isotactic regioregular EVOH polymers with 75 mol % ethylene were made from ROMP-hydrogenation-deprotection of a novel ROMP monomer, 3OBocCOE. DSC showed that regioregularity and isotacticity increased *T*_m_, χ_c_, and *T*_g_ in the ROMP-derived polymers, but WAXS data were less conclusive. Despite this, oxygen permeability was lower in the atactic sample compared to the isotactic sample. However, the atactic sample had a value of *P*_O2_ approaching that of commercial EVOH(44)-26 while maintaining a *P*_H2O_ value over 3 times lower. This shows that structural regularity and melt-processing together can furnish a monomaterial with competitive oxygen permeability but greatly improved water permeability, eliminating difficulties in recycling multilayer barrier packaging. In the future, more work could be done to investigate the crystal structure of the ROMP-derived polymers from drawn fibers to determine the nature of *P*_O2_ differences from intermolecular hydrogen-bonding, similar to that of Tashiro et al.^23^

## Data Availability

The data that support the findings of this study will be openly available in the Data Repository at the University of Minnesota (DRUM): https://doi.org/10.13020/qhy9-7196.

## Supporting Information Available

Experimental details, NMR spectra, NMR kinetics data, IR data, SEC data, TGA traces, additional 1D and 2D scattering data and figures, additional rheology tensile and gas barrier data (PDF)
POM video of EVOH(44)-26 (MP4)
POM video of PH3OHCOE-59: heating (MP4)
POM video of PH(*R*)3OHCOE-58: heating (MP4)
