# Supplementary material for: Regio- and Stereoregular EVOH Copolymers from ROMP as Designer Barrier Materials
Source: ACS Polym Au. 2024 Apr 11;4(3):208–13. doi: 10.1021/acspolymersau.4c00006 (PMC11177297; doi:10.1021/acspolymersau.4c00006)
Supplement: Supplementary file 1 — lg4c00006_si_001.pdf [file lg4c00006_si_001.pdf]

Regio- and stereoregular EVOH Copolymers from ROMP as Designer Barrier Materials

Claire E. Dingwell and *Marc A. Hillmyer**

Department of Chemistry, University of Minnesota, Minneapolis, Minnesota 55455, United States.

Corresponding Author: Marc A. Hillmyer, hillmyer@umn.edu

Table of Contents

Materials 3

Characterization 3-4

Synthetic Procedures 4-7

Processing Procedures 7

Figures and Tables 8-39

References 40

Materials

All chemicals and reagents were purchased from chemical suppliers and used without further purification, except *cis*-4-octene, which was distilled under argon prior to use. 3-hydroxycyclooctene (3OHCOE) was prepared using literature procedures.[1] Hydrogenation and deprotection procedures were followed from our previous study.[2] Anhydrous toluene was obtained from a solvent purification system (Pure Process Technology) for polymerization. Commercial EVOH(44)-26 was generously donated by the DOW Chemical Company.

Characterization

Nuclear magnetic resonance (NMR) spectroscopy: 1D- (^{1}H, ^{13}C) and 2D- (COSY, HSQC) spectra were recorded on either an AM-400, AV-500, or HD-500 Bruker Avance III spectrometer. Chemical shifts (δ values) are reported in parts per million and referenced to the residual solvent signal. For $CDCl_3$: ^{1}H singlet = 7.26, ^{13}C triplet = 77.16. For TFA-d: ^{1}H singlet = 11.50, ^{13}C quartet = 116.5. Characteristic splitting patterns due to spin-spin coupling are identified as follows: s = singlet, dd = doublet of doublets, dt = doublet of triplets, m = multiplet. All coupling constants are measured in hertz (Hz).

Fourier Transform Infrared (FT-IR) spectroscopy: Data was collected on a Bruker Alpha Platinum ATR FT-IR spectrometer with a platinum attenuated total reflectance (ATR) sampling module with a diamond crystal. Measurements were performed in transmittance mode averaged over 64 scans.

Size exclusion chromatography (SEC): THF SEC was performed using an Agilent 1260 series instrument (1.0 mL·min^{-1}, 25 °C). Separation was achieved using a single Tosoh Styragel guard column and three successive Tosoh Styragel columns (G6000, G4000, and G2000) packed with rigid 5 μm styrene-divinylbenzene particles. Number-average molecular weight (M_n), weight-average molecular weight (M_w), and dispersity ($Đ$) were determined using a Wyatt Technology DAWN Heleos II multiangle laser light scattering (MALS) detector and a Wyatt OPTILAB T-rEX refractive index detector (based on a 10-point calibration curve with polystyrene standards). HFIP SEC (0.05 M potassium trifluoroacetate in HFIP) was performed using an EcoSEC HLC-8240GPC series liquid chromatograph (0.35 mL·min^{-1}, 40 °C) fitted with a refractive index detector and two Tosoh TSKgel SuperAWM-H columns. Molar mass was determined by conventional calibration against poly(methyl methacrylate) (PMMA) standards.

Thermal analyses: Thermogravimetric analysis (TGA) was performed on a TA Instruments Q500 or a Mettler Toledo TGA under nitrogen atmosphere at a heating rate of 10 °C·min^{-1}. Scanning Calorimetry (DSC) was performed on a TA Instruments Discovery DSC calibrated with an indium standard using hermetically-sealed, aluminum T-zero pans. Scans were conducted at a heating rate of 10 °C·min^{-1}.

Tensile testing: Measurements performed on a Shimadzu Autograph AGS-X series instrument to conduct uniaxial extension measurements at room temperature with dog-bone shaped samples (3 mm width x 15 mm gauge length, variable thickness). The test speed was set to a ramp rate of 1 mm·min^{-1}. The data obtained were analyzed using the *Trapezium*

software and Microsoft excel. All were performed with 5 replicates per sample to break, and the values are reported as the average for each sample, excluding outliers.

Dynamic Mechanical Thermal Analysis (DMTA): Measurements were performed using a TA Instruments RSA-G2 analyzer with dog-bone shaped specimens (same dimensions as above) in a rectangular tension geometry. The axial force was set to 0.00 N (0.01 N sensitivity) after the sample was loaded. The sample equilibrated at room temperature for 2 min. The proportional force mode was set to force tracking to maintain an axial force that was at least 100% greater than the dynamic oscillatory force. The strain adjust was set to 30% with minimum and maximum strain values of 0.05 and 10% and minimum and maximum forces of 0.01 and 0.2 N, respectively; these settings prevented the sample from going outside the specified strain range. The sample was then heated to 200 °C at a rate of 5 $°C \cdot min^{-1}$ using a gas oven with a frequency of 1.0 Hz, a strain of 0.1%, and an axial force of 20.0 g.

Wide-angle X-ray scattering (WAXS): Polymer samples were mounted on a Teflon washer with Kapton tape. Experiments were conducted at the DuPont-Northwestern Dow Collaborative Access Team (DND-CAT) synchrotron research center 5-ID-D beamline of the Advanced Photon Source at the Argonne National Laboratory using a sample-to-detector distance of 8.5 m and a photon wavelength of $\lambda = 0.729$ Å. Two-dimensional (2D) scattering patterns were collected using a Rayonix area CCD detector. For PH3OHCOE-59, sample rerun was performed at the University of Minnesota on a Bruker-AXS Smart Apex-II using a Mo K(α) source.

Polarized Optical Microscopy (POM): Data was collected on an Olympus BX52 Microscope using a Linkam T95 temperature stage. Samples were heated at a rate of 10 $°C \cdot min^{-1}$ with a 4x objective. Time-lapse videos were created from images taken during the heat ramp to visualize sample changes throughout heating.

Oxygen Transmission Rate (OTR) Testing: Data was collected at the Clemson University Center for Flexible Packaging on a Mocon OxTran 2/21 Model H Instrument at 23°C with 0% relative humidity. Nitrogen was used as the carrier gas, and oxygen was used as the test gas (30 psi). WVTR was collected on a MOCON Permatran 3/33 Model G instrument at 38 °C with 90% relative humidity (35 psi).

Gas Chromatography Mass Spectrometry (GCMS): Mass spectra were obtained using an Agilent 5975 MSD at 70 eV. This was connected to Agilent 6953 gas chromatograph fitted with an Agilent HP-5 column (0.25 μm film thickness, 15 m length × 0.32 mm ID).

Synthetic Procedures

Synthesis of (Z)-tert-butyl cyclooct-2-en-1-yl carbonate[3] (**3OBocCOE**)

3-hydroxycyclooctene (3OHCOE) (10.3 g, 81.6 mmol), Di-tert-butyl decarbonate (19.6 g, 89.8 mmol), $Zn(OAc)_2 \cdot 2H_2O$ (1.8 g, 8.2 mmol), and DCM (82 mL) were added to a 250 mL round-bottom flask equipped with a Teflon-coated stir bar and reflux condenser. The reaction was heated to reflux for 24 h. After cooling to room temperature, the reaction mixture was diluted

with water and extracted 3 times with DCM. The combined organic layers were washed with brine, dried over $MgSO_4$ and concentrated. The resulting colorless oil was purified by column chromatography (silica gel, 1:3 EtOAc/hexane). 14.4 g obtained (78% yield).

3OBocCOE: ^{1}H-NMR (500 MHz, $CDCl_3$): δ (ppm) = 5.64-5.72 (m, 1H), 5.50-5.56 (m, 1H), 5.41-5.50 (m, 1H), 2.18-2.32 (m, 1H), 2.07-2.17 (m, 1H), 1.92-2.02 (m, 1H), 1.64-1.74 (m, 1H), 1.51-1.63 (m, 5H), 1.49 (s, 9H), 1.34-1.44 (m, 1H). ^{13}C-NMR (500 MHz, $CDCl_3$): δ (ppm) = 153.29, 130.72, 129.93, 82.07, 75.42, 35.18, 28.98, 27.99, 26.52, 26.00, 23.47. GC-MS (50 °C/2.0 min/20 °C·min^{-1}/250 °C) t_R = 7.13 min; MS [70 eV, m/z (rel int)]: 226 (M+), 170 (M+ - C_4H_8, *t*butyl), 108 (M+ - OBoc).

Caution! When heating a reaction to reflux with a condenser, a water recirculator should be used to prevent flooding.

Synthesis of (R)-(Z)-tert-butyl cyclooct-2-en-1-yl carbonate[1,4] [(***R***)**3OBocCOE**]

A 1L Schenk flask was charged with $Pd_2(dba)_3$·$CHCl_3$ (1.38 g, 1.33 mmol), (*R*,*R*)-DACH-phenyl Trost ligand (2.75 g, 3.98 mmol), and 250 mL degassed DCM under an inert atmosphere. The reaction stirred at room temperature for 30 min until the color turned from deep brown to orange. Meanwhile, 3OBocCOE (15.0 g, 66.4 mmol) was placed in a 250 mL Schlenk flask with 20 mL DCM. Tetrahexylammonium bromide (1.8 g, 4.1 mmol), sodium benzenesulfinate (23.0 g, 140 mmol), and 80 mL water were placed in another 250 mL Schlenk flask. Both flasks were degassed by 3 freeze-pump-thaw cycles. The solution containing 3OBocCOE was added to the 1L reaction flask. After 30 min, the aqueous solution was added. The reaction stirred for 72 h. The reaction was opened to air and diluted with water. The aqueous later was extracted 3 times with DCM. The combined organic layers were washed with brine, dried over magnesium sulfate, and concentrated. The resulting colorless oil was purified by column chromatography (silica gel, 1:9 EtOAc/hexane). 4.35 g (58 % yield) colorless oil was obtained. NMR spectra were indistinguishable from (±)-3OBocCOE.

Determination of enantiomeric excess by Mosher ester synthesis

Adapted from Hoye *et al.*,[5] (*R*)3OBocCOE (63.1 mg, 0.279 mmol) and THF (2 mL) were placed in a dram vial and stirred. Sodium methoxide (25% in methanol, 255 mg, 1.18 mmol) was added to the vial. The reaction was monitored by TLC. After reaction completion, the reaction was quenched with HCl (10 vol % in H_2O) until the pH was acidic. The aqueous layer was extracted 3 times with ether. The combined organic layers were dried over magnesium sulfate and concentrated. The resulting (*R*)3OHCOE (34 mg, 0.27 mmol) was added to another vial with stir bar. (*R*)-(+)-α-Methoxy-α-trifluoromethylphenylacetic acid (191 mg, 0.816 mmol), *N*,*N'*-Dicyclohexylcarbodiimide (96.4 mg, 0.468 mmol), 4-Dimethylaminopyridine (75 mg, 0.61 mmol) and 2 mL DCM were added to the vial. The reaction mixture was stirred for 48 h until completion was observed by TLC. The reaction was filtered through cotton to remove insoluble solids and the filtrate was concentrated. The filtrate was purified by column chromatography (silica gel, 1:4 EtOAc, hexane).

This process was repeated on the racemic mixture to determine separation of (R) and (S) compounds. The peak corresponding to the (R) enantiomer was determined to be at 5.56 ppm (^{1}H-NMR, $CDCl_3$), while the (S) enantiomer was determined to be at 5.41 ppm. Based on these determinations, it was found that no detectable (S) enantiomer was present in (R)3OBocCOE. Enantiomeric excess can be calculated using **Equation (1)**.

$$\% \, ee = \frac{([R] - [S])}{([R] + [S])} \tag{1}$$

Conversion Study of 3OBocCOE

3OBocCOE was filtered through basic alumina with hexanes and concentrated. 3OBocCOE (501 mg, 2.22 mmol) and 1.7 mL toluene-*d*8 were added to a 2-neck round-bottom flask with a Teflon-coated stir bar. The solution was degassed by 3 freeze-pump-thaw cycles. Meanwhile, 4 mL toluene-*d*8 was degassed by sparging with argon for 30 min. 1.2 mg G2 was added to a vial, and the vial was evacuated and refilled with argon. 0.72 mL degassed toluene-*d*8 was added to the vial. 0.3 mL (0.5 mg, 0.0006 mmol) of this solution were added to the reaction flask. 0.5 mL of this solution were immediately added to an NMR tube under inert atmosphere. The NMR tube was quickly placed in a The Bruker Avance III HD nanobay AM-400 at 40 °C. Proton scans were taken at 9.85 min, 20.10 min, 30.04 min, 40.00 min, 50.00 min, 60.00 min, and 70.21 min. After 70.21 min, the reaction had reached full conversion and the reaction mixture was discarded. To calculate conversion, the integrations of the monomer and polymer olefins was determined in the ^{1}H-NMR spectrum (toluene-*d*8). The ratio of monomer to polymer was determined using these integration values and the % conversion was calculated.

General Polymerization Procedure

3OBocCOE (or (R)3OBocCOE) (10.01 g, 44.3 mmol, 3900 equiv) was filtered through basic alumina with hexanes and concentrated. Monomer, *cis*-4-octene (9.9 mg, 0.088 mmol, 7.8 equiv), and 39 mL toluene were added to a 250 mL Schlenk flask with a Teflon-coated stir bar. The reaction mixture was degassed 3 times with freeze-pump-thaw cycles. The flask was refilled with argon, and the reaction mixture was heated to 40 °C. G2 (9.6 mg, 0.011 mmol, 1 equiv) was added to a small vial, and the vial was evacuated and refilled with argon 3 times. G2 was dissolved in 1 mL toluene, then the solution was added to the reaction mixture. After 2 h, the reaction mixture was quenched with 1 mL ethyl vinyl ether and stirred for 30 min at room temperature. The reaction mixture was diluted with DCM, then the solution was precipitated in MeOH with trace BHT. The resulting polymer was dried in a vacuum oven at 60 °C overnight.

P3OBocCOE: ^{1}H-NMR (500 MHz, $CDCl_3$): δ (ppm) = 5.69 (dt, J = 15.3, 7.2 Hz, 1H), 5.36 (dd, J = 15.3, 7.5 Hz, 1H), 4.90 (dt, J = 6.9, 6.9 Hz, 1H), 1.99 (dt, J = 6.9, 6.9 Hz, 2H), 1.62-1.74 (m, 1H), 1.49-1.56 (m, 1H), 1.47 (s, 9H), 1.22-1.39 (m, 6H). ^{13}C-NMR (500 MHz, $CDCl_3$): δ (ppm) = 153.21, 134.86, 128.34, 81.75, 78.38, 34.69, 32.26, 29.01, 28.96, 27.98, 25.23.

P(*R*)3OBocCOE: ^{1}H-NMR (500 MHz, $CDCl_3$): δ (ppm) = 5.70 (dt, *J* = 15.3, 7.4 Hz, 1H), 5.36 (dd, *J* = 15.5, 7.8 Hz, 1H), 4.91 (dt, *J* = 6.9, 7.0 Hz, 1H), 1.99 (dt, *J* = 7.0, 7.0 Hz, 2H), 1.60-1.73 (m, 1H), 1.49-1.53 (m, 1H), 1.47 (s, 9H), 1.32-1.39 (m, 2H), 1.23-1.32 (m, 4H). ^{13}C-NMR (500 MHz, $CDCl_3$): δ (ppm) = 153.22, 134.88, 128.35, 81.76, 78.40, 34.70, 32.27, 28.97, 27.99, 25.24.

PH3OBocCOE: ^{1}H-NMR (500 MHz, $CDCl_3$): δ (ppm) = 4.53-4.68 (m, 1H), 1.50-1.59 (m, 4H), 1.48 (s, 9H), 1.18-1.36 (m, 10H). ^{13}C-NMR (500 MHz, $CDCl_3$): δ (ppm) = 153.80, 81.55, 77.87, 34.43, 29.63, 27.98, 25.49. FTIR (neat, cm^{-1}) = 3215, 2928, 2856, 1733, 1458, 1393, 1367, 1275, 1251, 1159, 1120, 1091, 1036, 957, 844, 791, 748, 723, 616, 563, 462, 433.

PH(*R*)3OBocCOE: ^{1}H-NMR (500 MHz, $CDCl_3$): δ (ppm) = 4.54-4.69 (m, 1H), 1.50-1.60 (m, 4H), 1.48 (s, 9H), 1.21-1.34 (m, 10H). FTIR (neat, cm^{-1}) = 2979, 2926, 2854, 1733, 1468, 1393, 1367, 1275, 1250, 1160, 1119, 1090, 1035, 1013, 996, 951, 884, 837, 790, 747, 723, 560, 501, 463, 436.

PH3OHCOE: ^{1}H-NMR (500 MHz, $CDCl_3$): δ (ppm) = 5.16-5.28 (m, 1H), 1.64-1.90 (m, 4H), 1.24-1.50 (m, 10H). ^{13}C-NMR (500 MHz, $CDCl_3$): δ (ppm) = 85.99, 36.52, 32.01, 27.86. FTIR (neat, cm^{-1}) = 3320, 2928, 2849, 1736, 1718, 1577, 1465, 1368, 1350, 1281, 1132, 1068, 1034, 855, 813, 722, 602, 559, 426.

PH(*R*)3OHCOE: ^{1}H-NMR (500 MHz, $CDCl_3$): δ (ppm) = 5.20-5.27 (m, 1H), 1.70-1.85 (m, 4H), 1.30-1.52 (m, 10H). ^{13}C-NMR (500 MHz, $CDCl_3$): δ (ppm) = 85.99, 36.52, 32.01, 27.86. FTIR (neat, cm^{-1}) = 3310, 2919, 2848, 1736, 1581, 1462, 1368, 1352, 1280, 1133, 1068, 1033, 945, 881, 854, 813, 791, 762, 722, 613, 553, 460, 447, 435, 417.

Processing Procedure

Polymer powder was melt-pressed to form thin films using a Wabash Genesis press 15 °C above their respective T_m for 3 min at 15,000 psig (103 MPa), then cooled to room temperature at 25 °C·min^{-1} inside the press. Samples were annealed for 2 hours under an inert atmosphere.

Figures

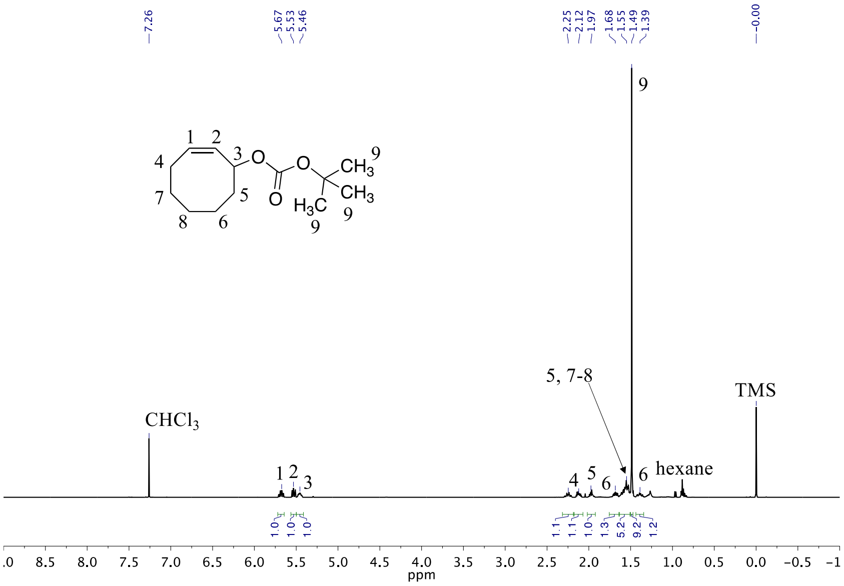

Figure S1. Representative ^{1}H-NMR ($CDCl_3$) of 3OBocCOE.

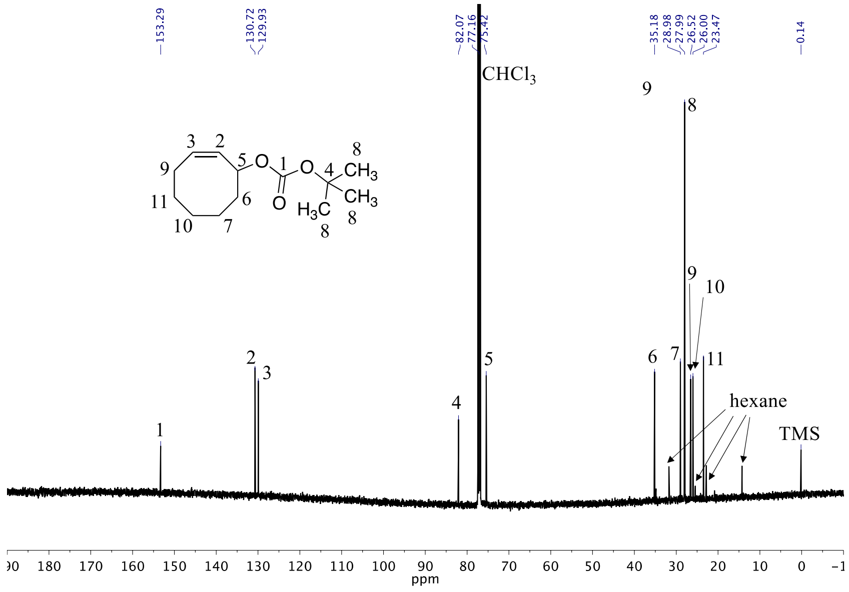

Figure S2. Representative ^{13}C-NMR ($CDCl_3$) of 3OBocCOE.

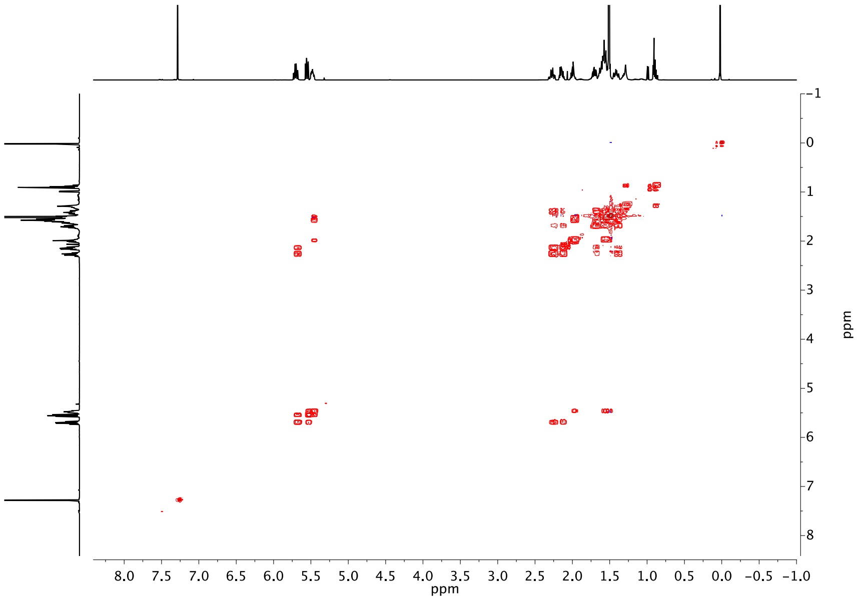

Figure S3. Representative COSY-NMR ($CDCl_3$) of 3OBocCOE.

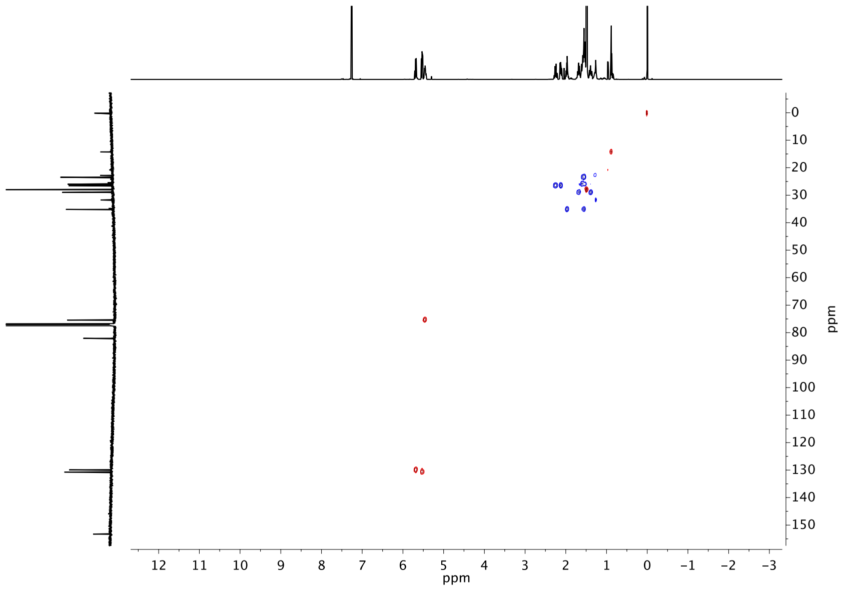

Figure S4. Representative HSQC-NMR ($CDCl_3$) of 3OBocCOE.

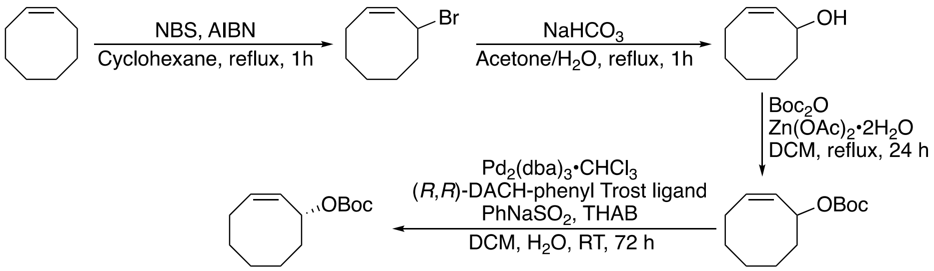

Figure S5. Synthesis of 3OBocCOE and following kinetic resolution through palladium catalyzed substitution of (*S*)-3OBocCOE.

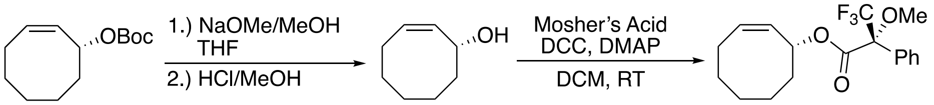

Figure S6. Mosher ester synthesis of (*R*)3OBocCOE after kinetic resolution.

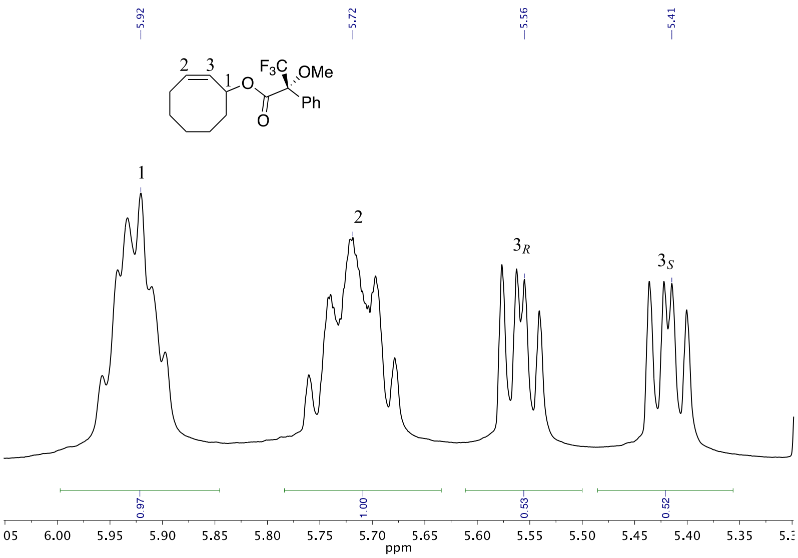

Figure S7. ^{1}H-NMR ($CDCl_3$) of the Mosher's Ester derivative of racemic 3OBocCOE. The olefin region is highlighted to better show the *R* and *S* peaks corresponding to both enantiomers.

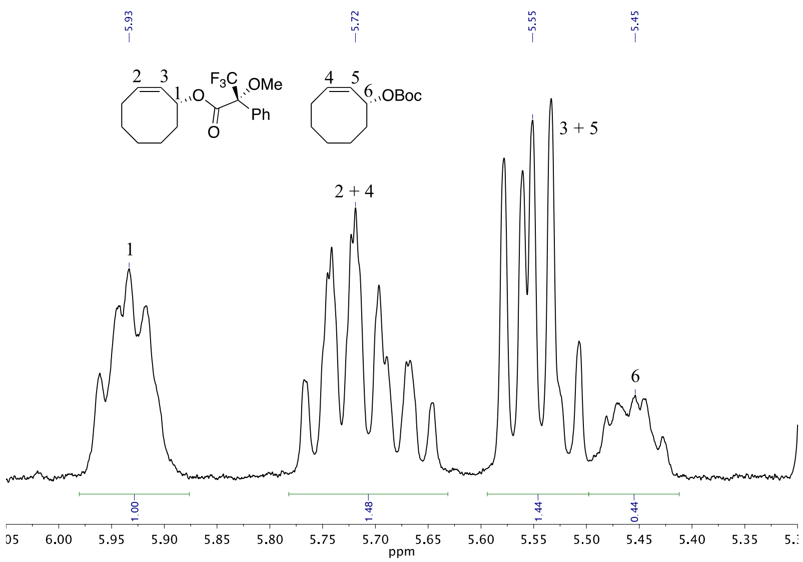

Figure S8. ^{1}H-NMR ($CDCl_3$) of the Mosher's Ester derivative of (*R*)3OBocCOE used for PH(*R*)3OBocCOE-58. Incomplete deprotection before esterification led to (*R*)3OBocCOE contamination.

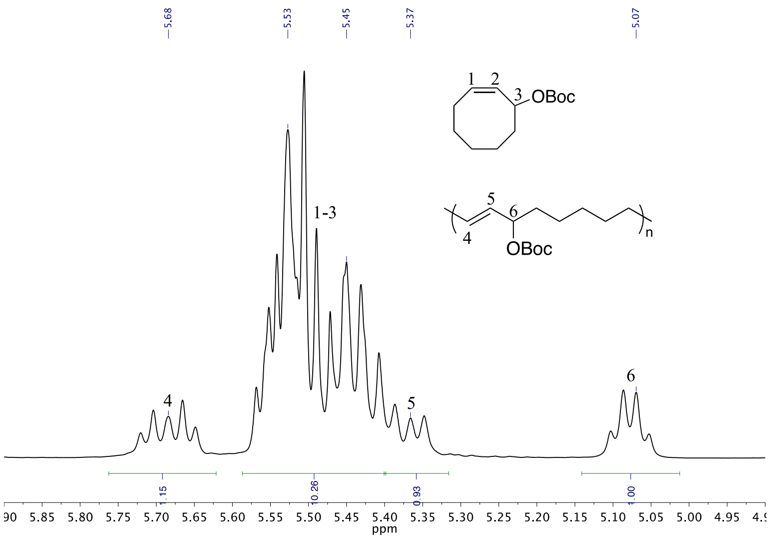

Figure S9. ^{1}H-NMR (toluene-*d*8) of 3OBocCOE kinetic experiment (10 min) in the olefin region. Monomer peaks are shown by numbers 1-3, and polymer peaks are shown by numbers 4-6.

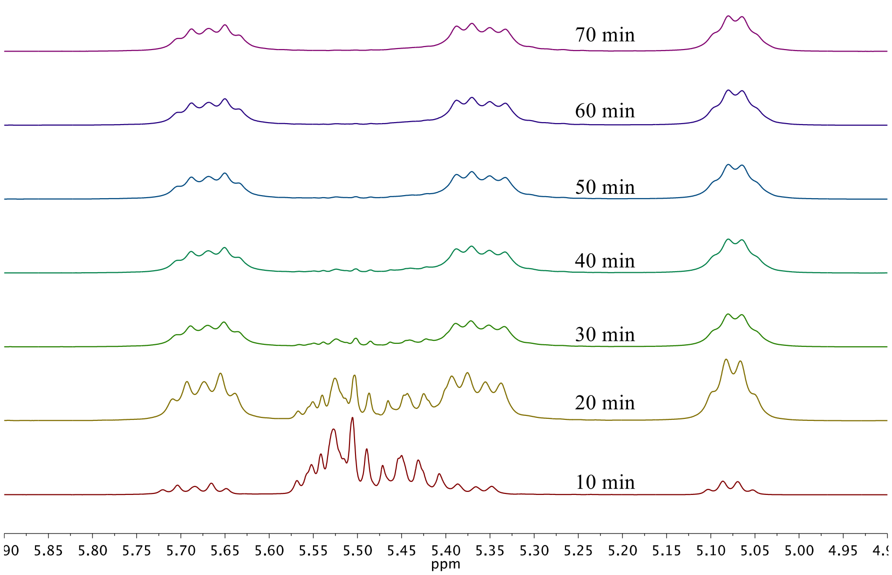

Figure S10. Overlay of all ^{1}H-NMR spectra (toluene-*d*8) from 10-70 minutes.

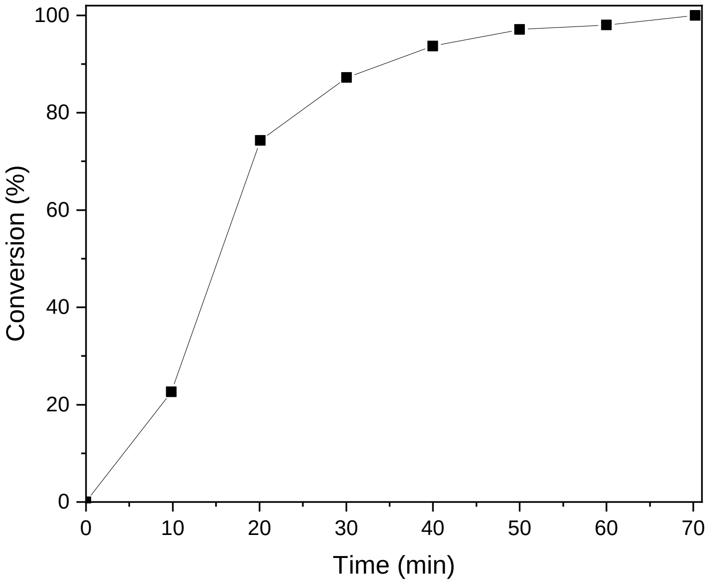

Figure S11. Conversion (%) of monomer to polymer vs. time, calculated from ^{1}H-NMR (toluene-*d*8) integrations.

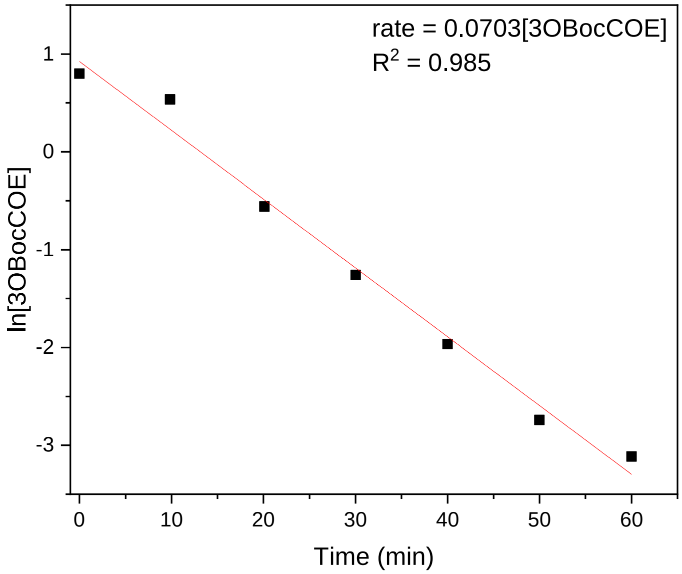

Figure S12. First order kinetic plot of 3OBocCOE polymerized with G2 in toluene-*d*8.

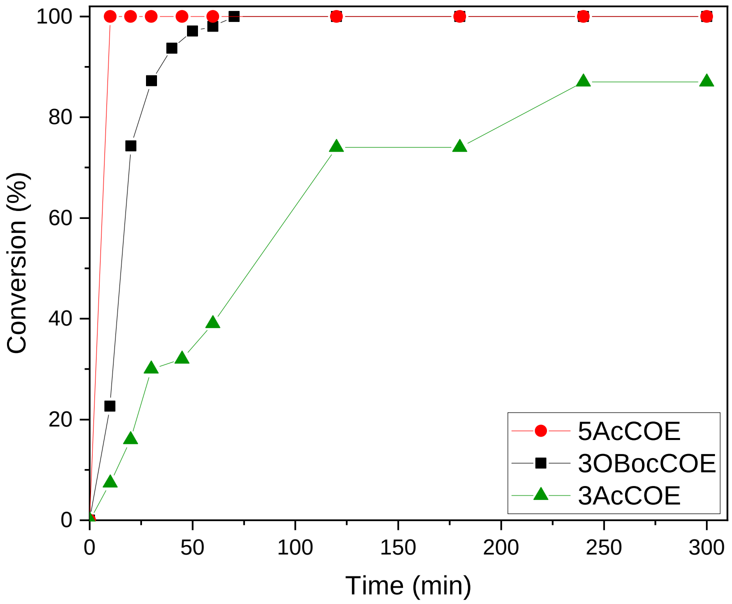

Figure S13. Conversion (%) vs. time (min) of 5AcCOE and 3AcCOE compared to 3OBocCOE (toluene-*d*8).

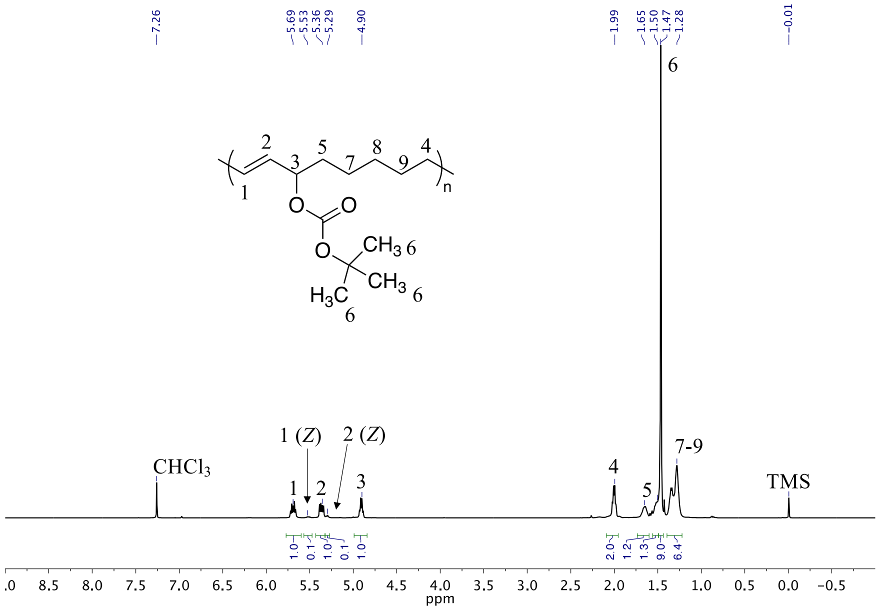

Figure S14. ^{1}H-NMR ($CDCl_3$) of atactic P3OBocCOE.

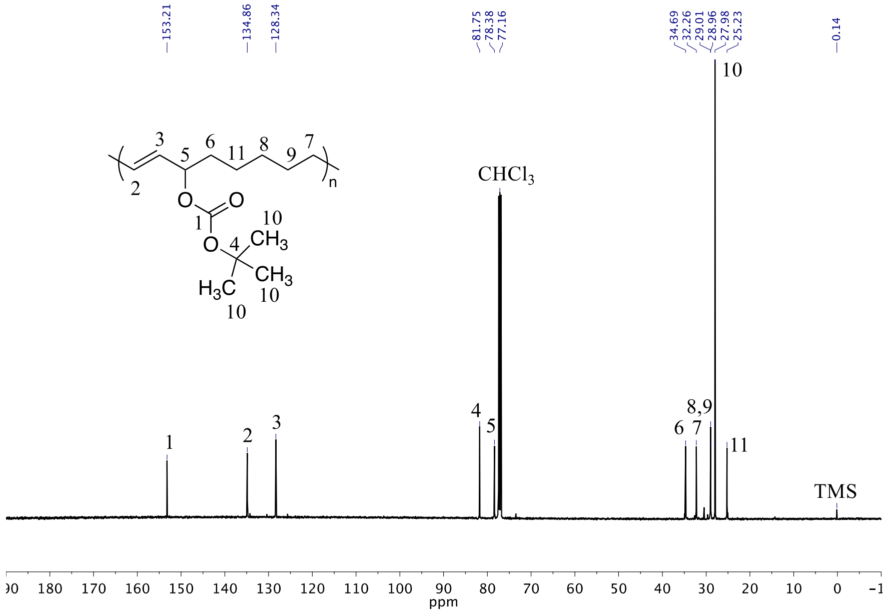

Figure S15. ^{13}C-NMR ($CDCl_3$) of atactic P3OBocCOE.

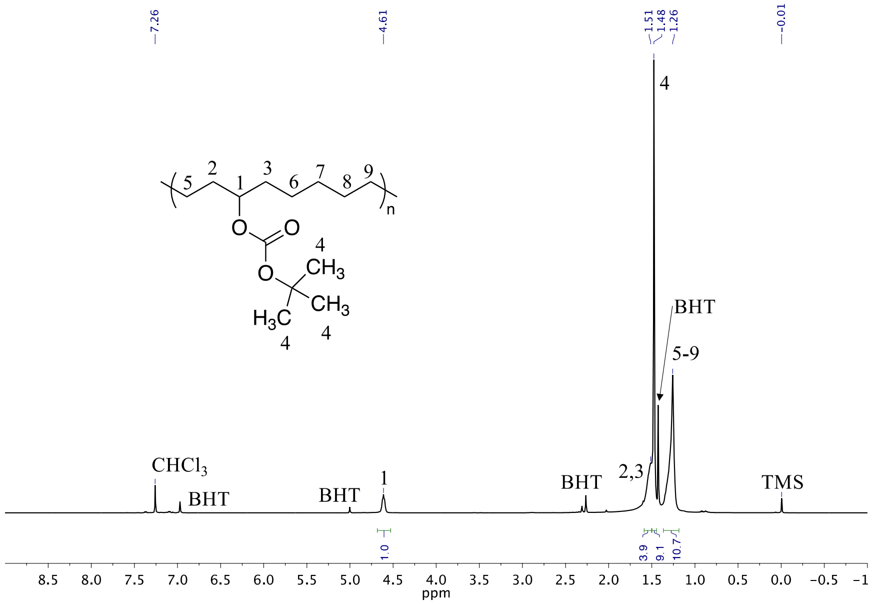

Figure S16. ^{1}H-NMR ($CDCl_3$) of atactic PH3OBocCOE.

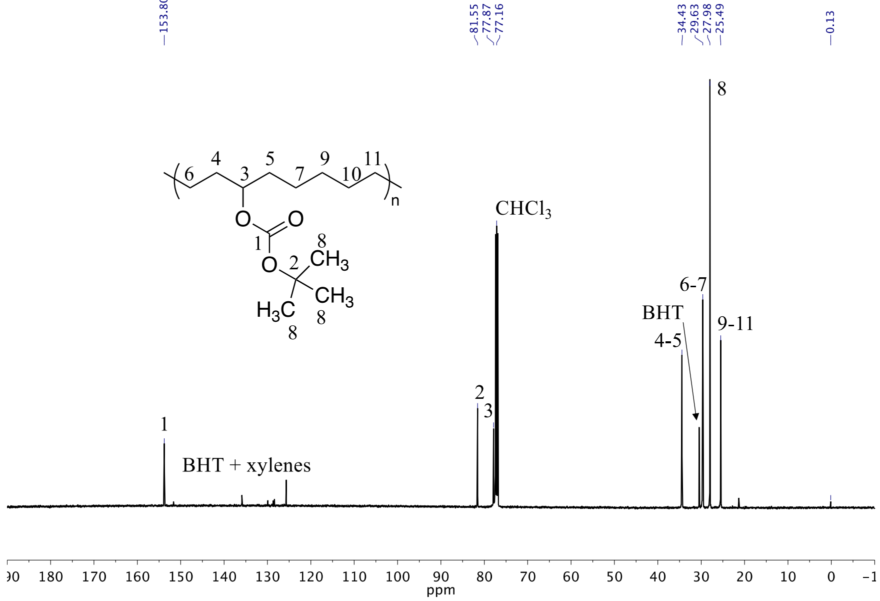

Figure S17. ^{13}C-NMR ($CDCl_3$) of atactic PH3OBocCOE.

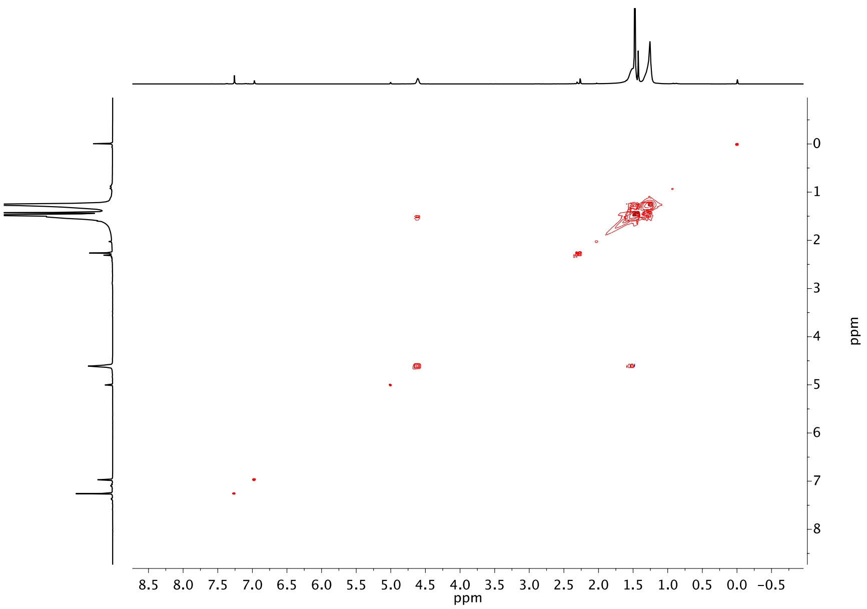

Figure S18. COSY-NMR ($CDCl_3$) of atactic PH3OBocCOE.

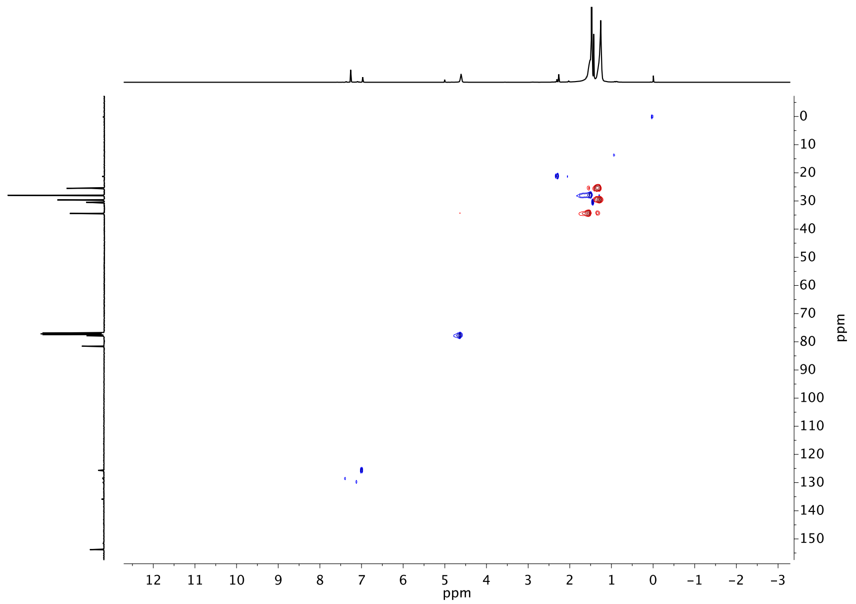

Figure S19. HSQC-NMR ($CDCl_3$) of atactic PH3OBocCOE.

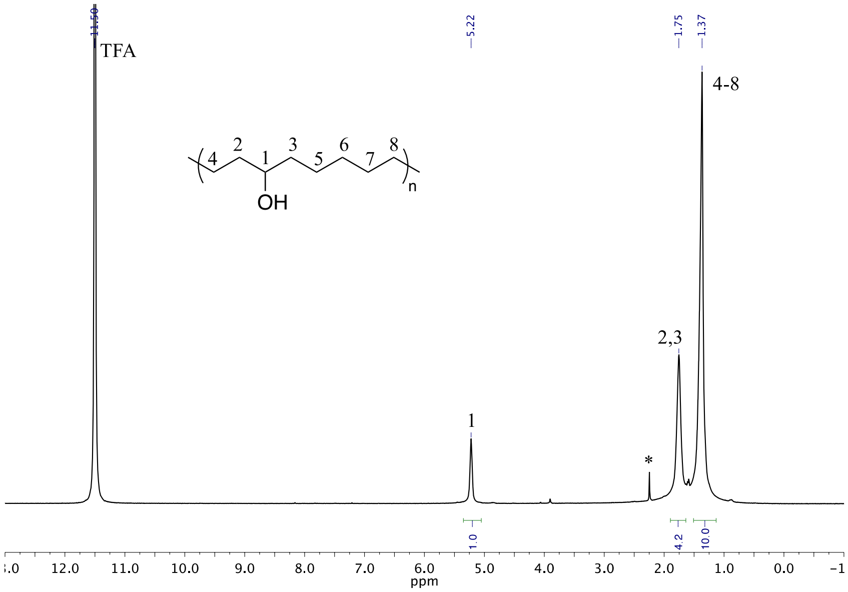

Figure S20. ^{1}H-NMR (TFA-*d*) of atactic PH3OHCOE-59. * indicates unknown impurity from HFIP solvent.

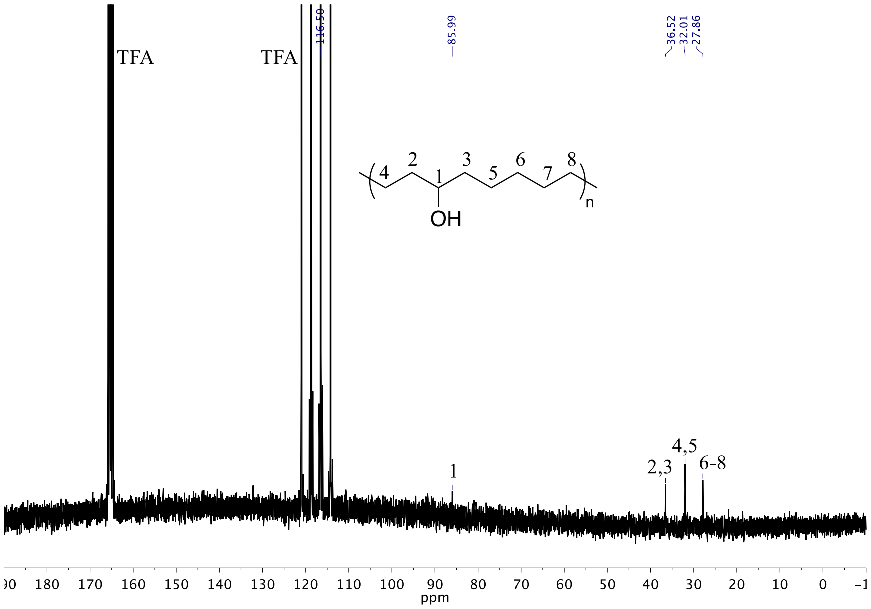

Figure S21. ^{13}C-NMR (TFA-*d*) of atactic PH3OHCOE-59.

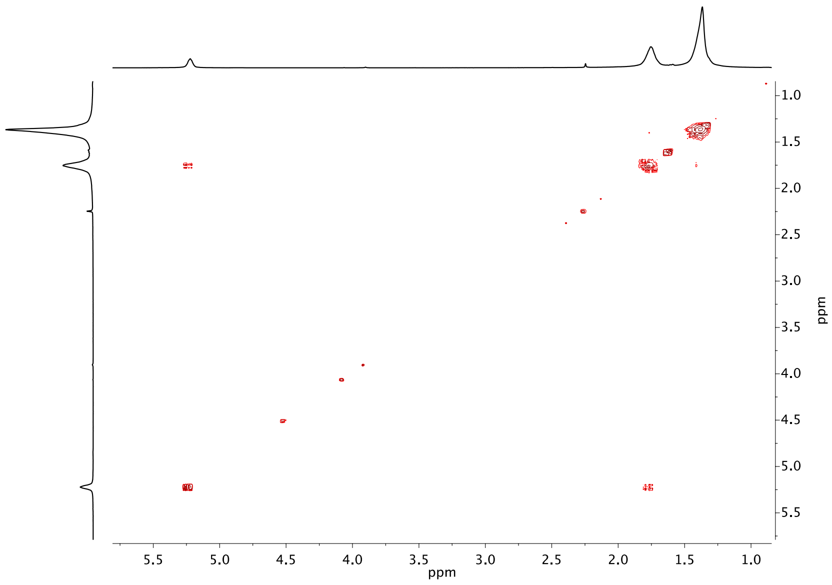

Figure S22. COSY-NMR (TFA-*d*) of atactic PH3OHCOE-59.

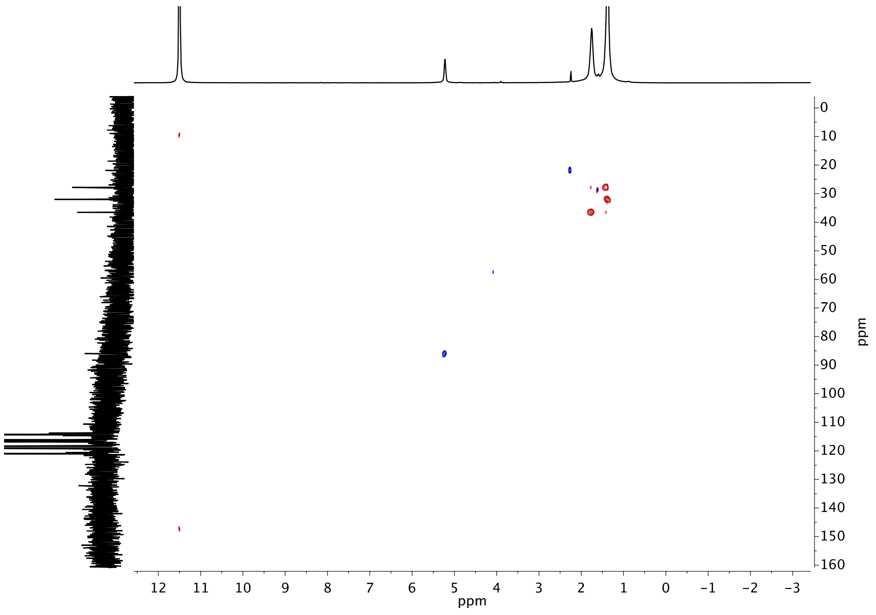

Figure S23. HSQC-NMR (TFA-*d*) of atactic PH3OHCOE-59.

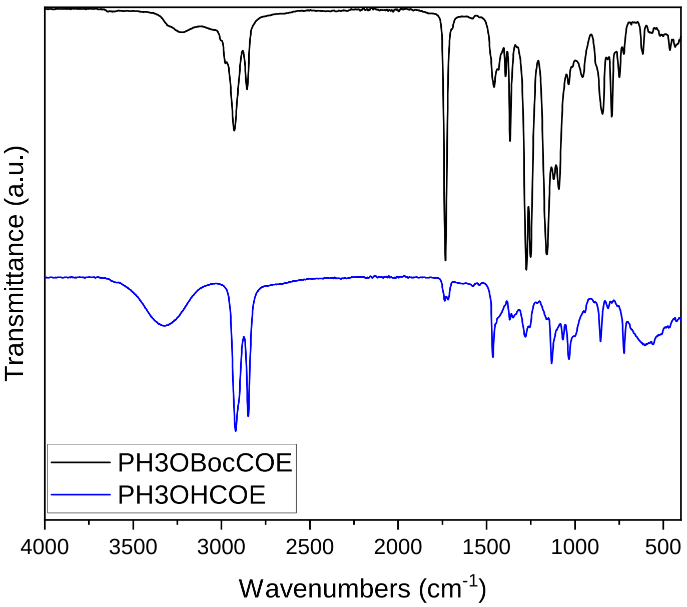

Figure S24. FT-IR of atactic PH3OBocCOE and PH3OHCOE-59 after deprotection.

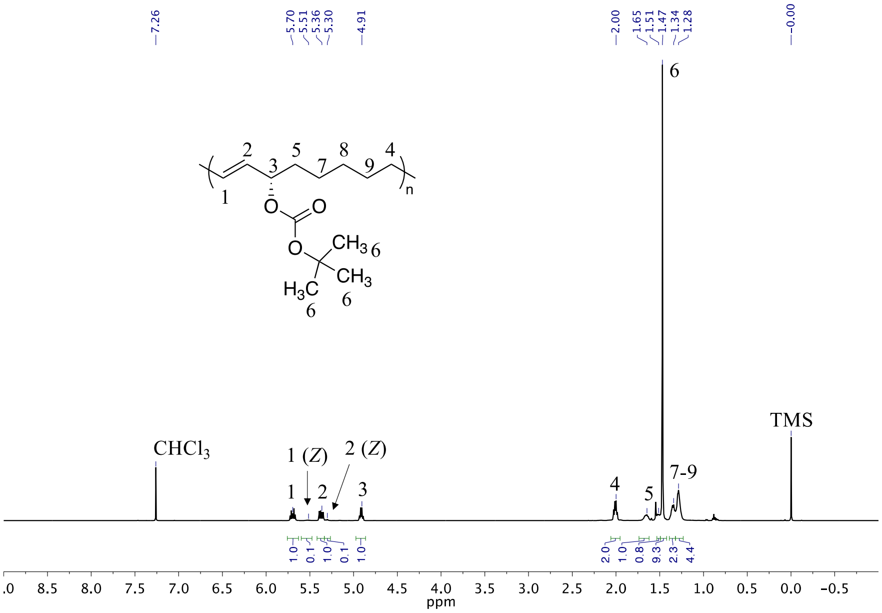

Figure S25. ^{1}H-NMR ($CDCl_3$) of P(*R*)3OBocCOE (precursor to PH(*R*)3OHCOE-58).

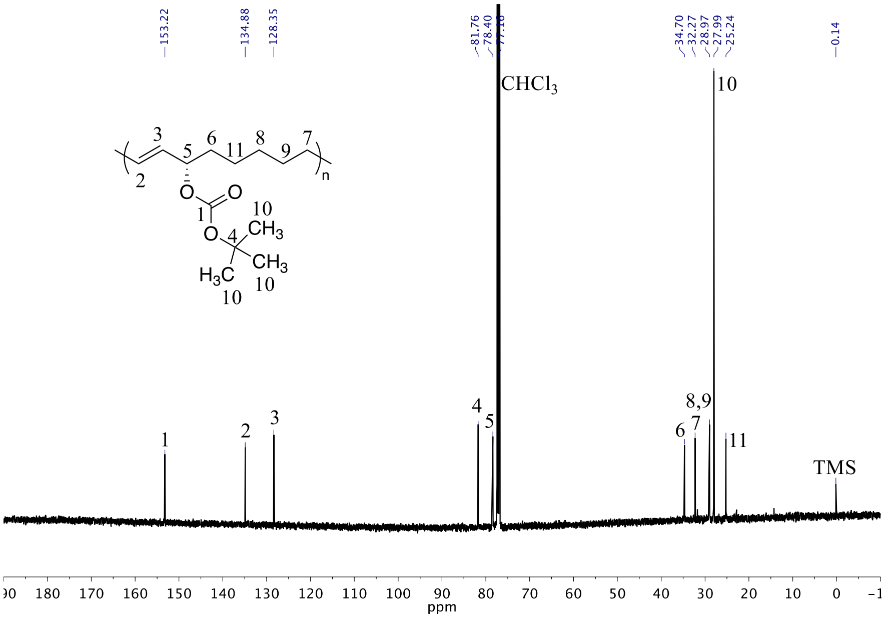

Figure S26. ^{13}C-NMR (CDCl$_3$) of P(*R*)3OBocCOE (precursor to PH(*R*)3OHCOE-58).

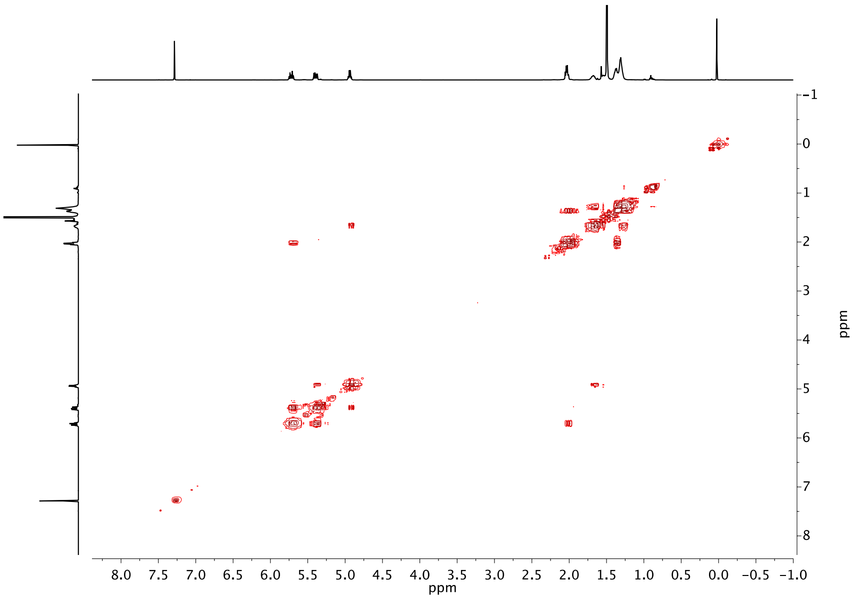

Figure S27. COSY-NMR (CDCl$_3$) of P(*R*)3OBocCOE (precursor to PH(*R*)3OHCOE-58).

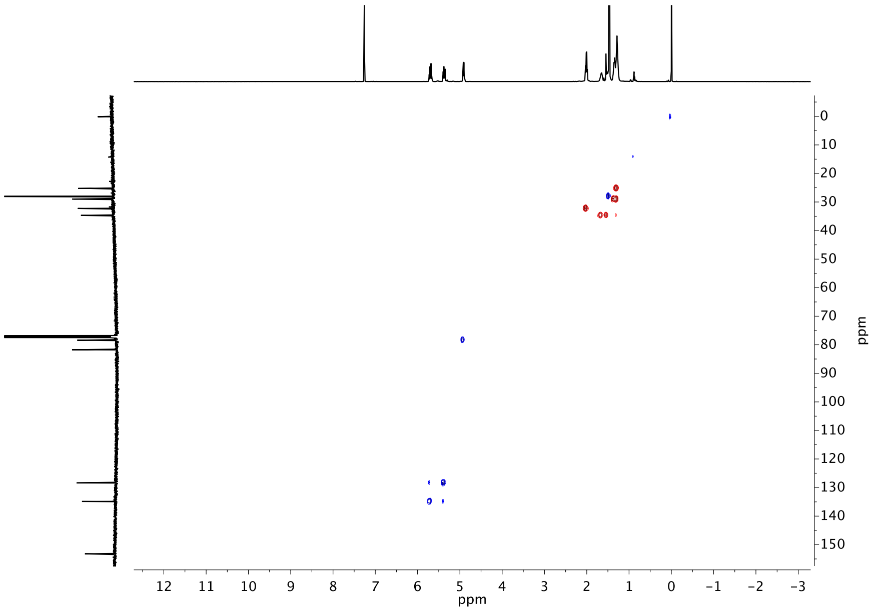

Figure S28. HSQC-NMR ($CDCl_3$) of P(*R*)3OBocCOE (precursor to PH(*R*)3OHCOE-58).

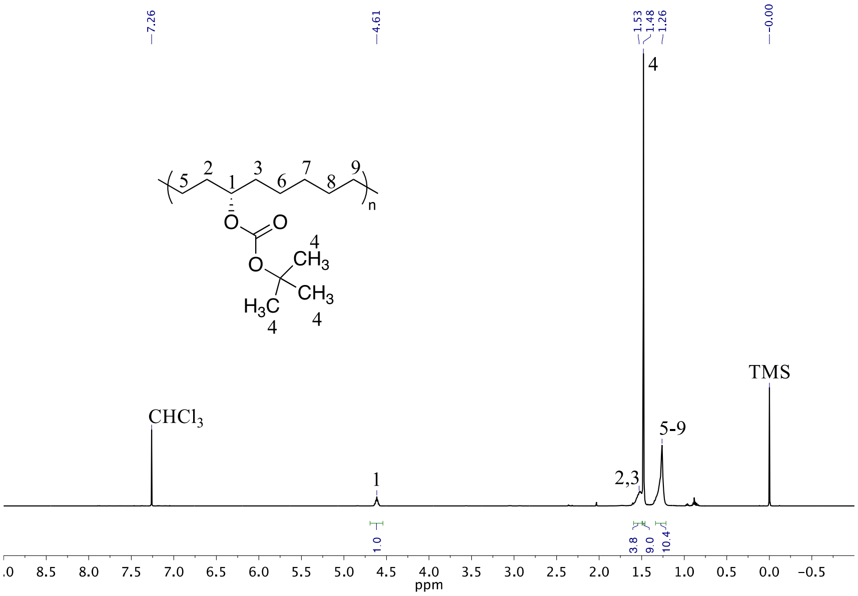

Figure S29. ^{1}H-NMR ($CDCl_3$) of PH(*R*)3OBocCOE (precursor to PH(*R*)3OHCOE-58).

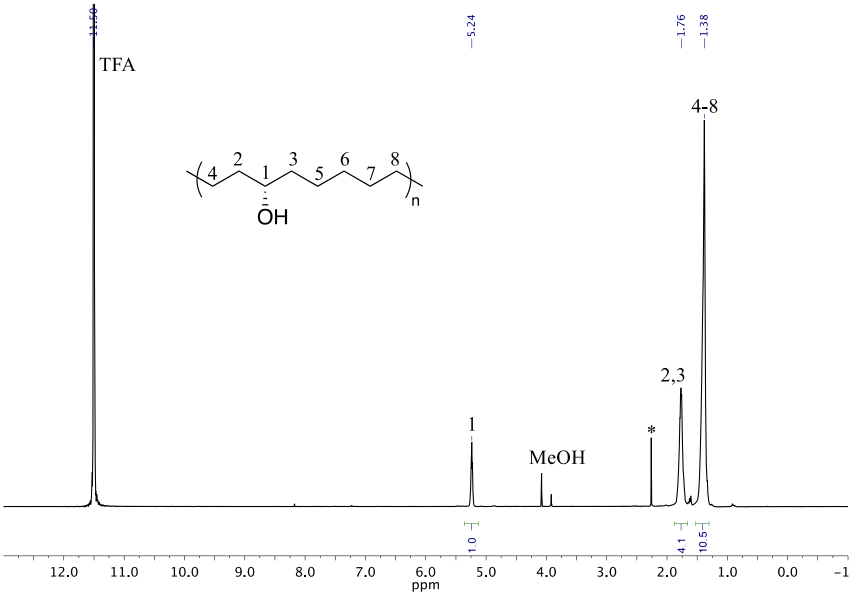

Figure S30. ^{1}H-NMR (TFA-*d*) of PH(*R*)3OHCOE-58. * indicates unknown impurity from HFIP solvent.

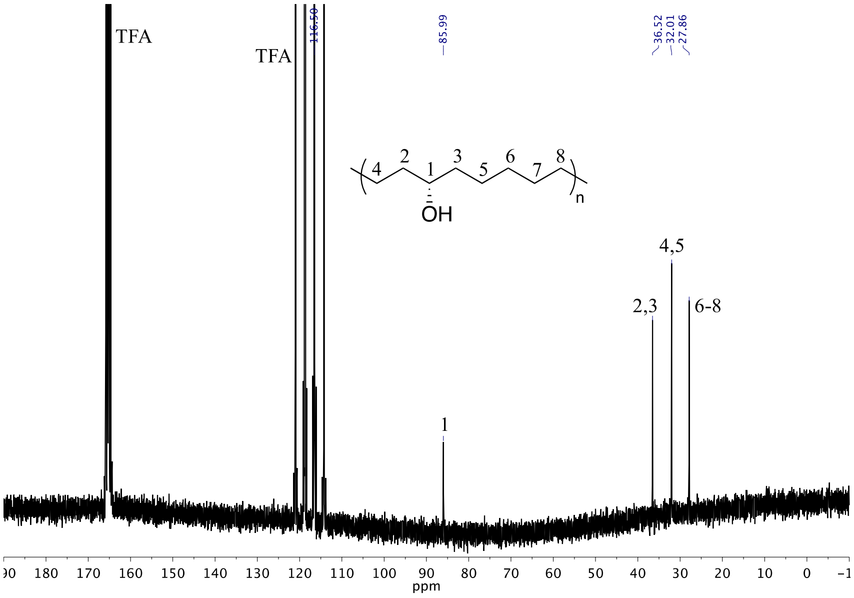

Figure S31. ^{13}C-NMR (TFA-*d*) of PH(*R*)3OHCOE-58.

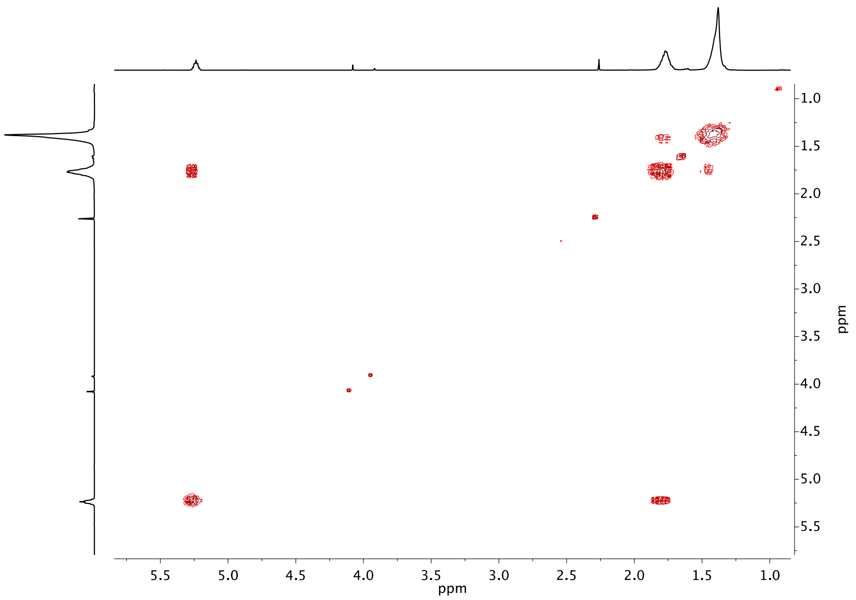

Figure S32. COSY-NMR (TFA-*d*) of PH(*R*)3OHCOE-58.

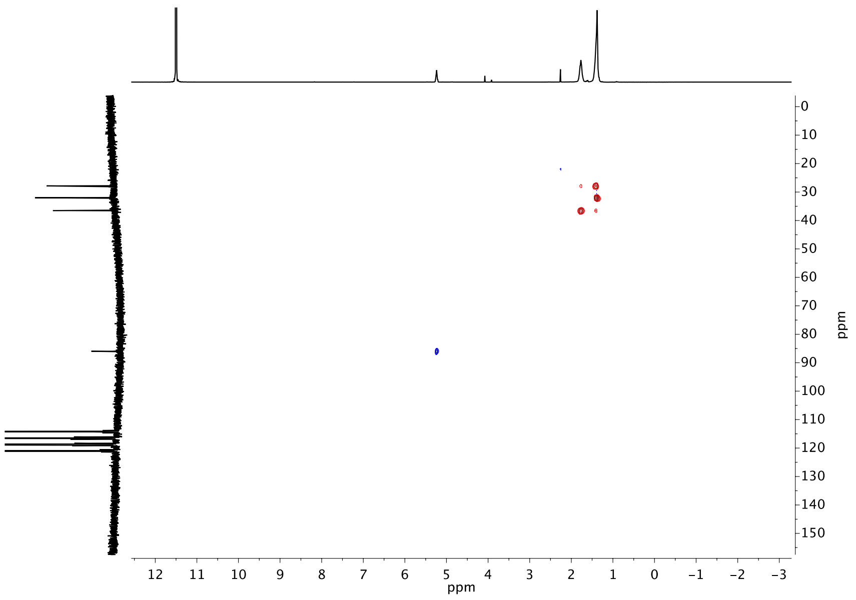

Figure S33. HSQC-NMR (TFA-*d*) of PH(*R*)3OHCOE-58.

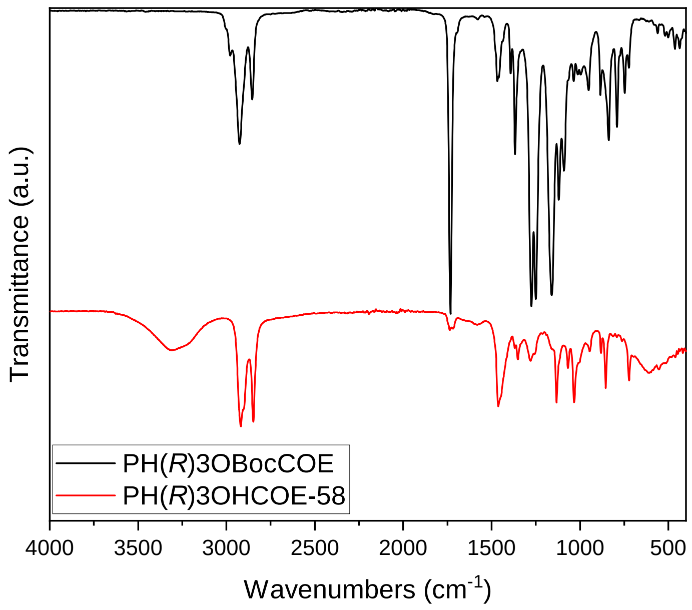

Figure S34. FT-IR of atactic PH(*R*)3OBocCOE and PH(*R*)3OHCOE-58 after deprotection.

Table S1. ROMP of 3OBocCOE and (*R*)3OBocCOE and characterization of resulting polymers.

	[M]:[CTA]:[G2]	%*ee*$_{monomer}$ [a]	Isolated Yield	*E*, HT (%)[b]
P3OBocCOE-59	3900:8:1	rac.	84	92, >99
P(*R*)3OBocCOE-58	3700:15:1	>99%	77	91, >99
	$M_{n,\,theo}$ (kg·mol^{-1})[c]	M_n (kg·mol^{-1})[d]	M_w (kg·mol^{-1})[d]	*Đ*[d]
P3OBocCOE-59	100	120	182	1.5
P(*R*)3OBocCOE-58	50	78	172	2.2

[a]Determined by Mosher's ester synthesis of corresponding monomer using ^{1}H-NMR integrations of *R* and *S* peaks (Figure S.X). %*ee* = $\{([H]_{BR}-[H]_{BS})/([H]_{BR}+[H]_{BS})\}\cdot 100$. [b]Determined by ^{1}H-NMR olefin integrations. [c]Calculated by $M_{n,theo}$ = $MW_{repeat\ unit}\cdot\{[M]/([CTA]+[G2])\}$. [d]Determined by THF-SEC (1.0 mL·min^{-1}, 25 °C) using MALS analysis.

Table S2. HFIP-SEC analysis of polymers from **Scheme 1.**

	M_n (kg·mol^{-1})	M_w (kg·mol^{-1})	$Đ$
PH5OHCOE-78	78	175	2.3
PH3OHCOE-59	59	86	1.5
PH(*R*)3OHCOE-58	58	94	1.6
EVOH(44)-26	26	69	2.6

Data obtained from HFIP-SEC (0.35 mL·min^{-1}, 40 °C) against PMMA standards.

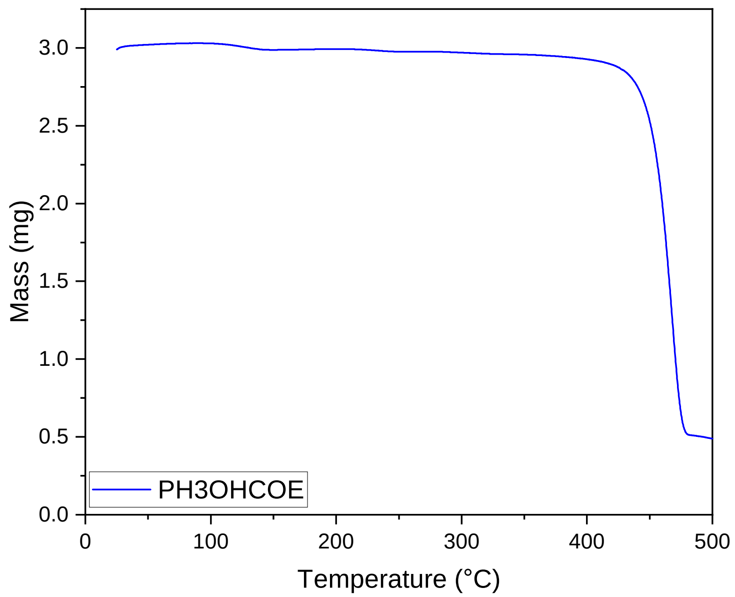

Figure S35. TGA trace of PH3OHCOE-59 (10 °C·min^{-1}).

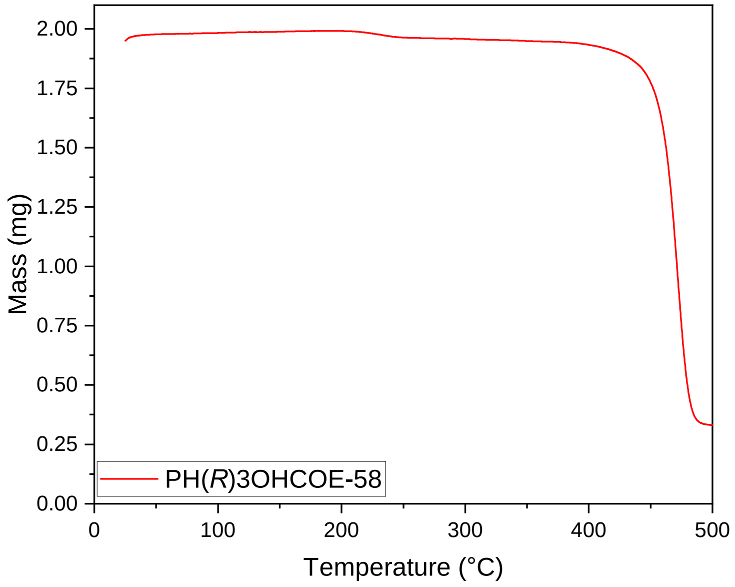

Figure S36. TGA trace of PH(*R*)3OHCOE-58 (10 °C·min^{-1}).

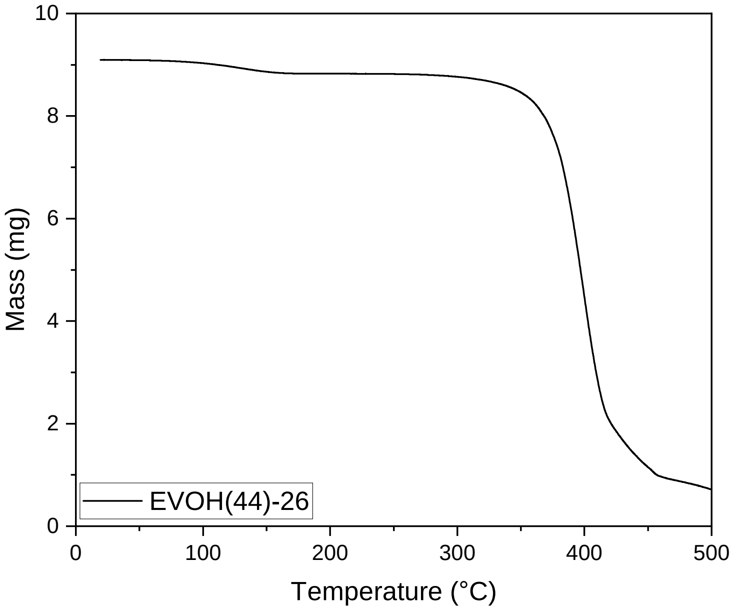

Figure S37. TGA trace of EVOH(44)-26 (10 °C·min^{-1}).

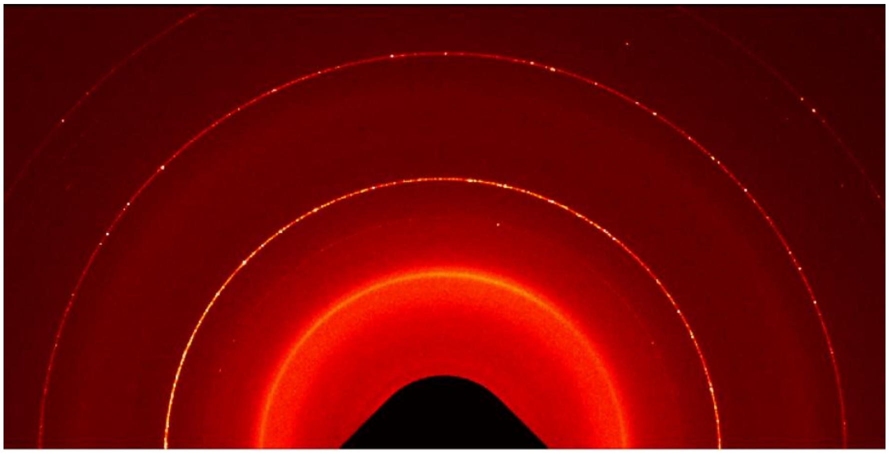

Figure S38. 2D WAXS image of PH3OHCOE-59.

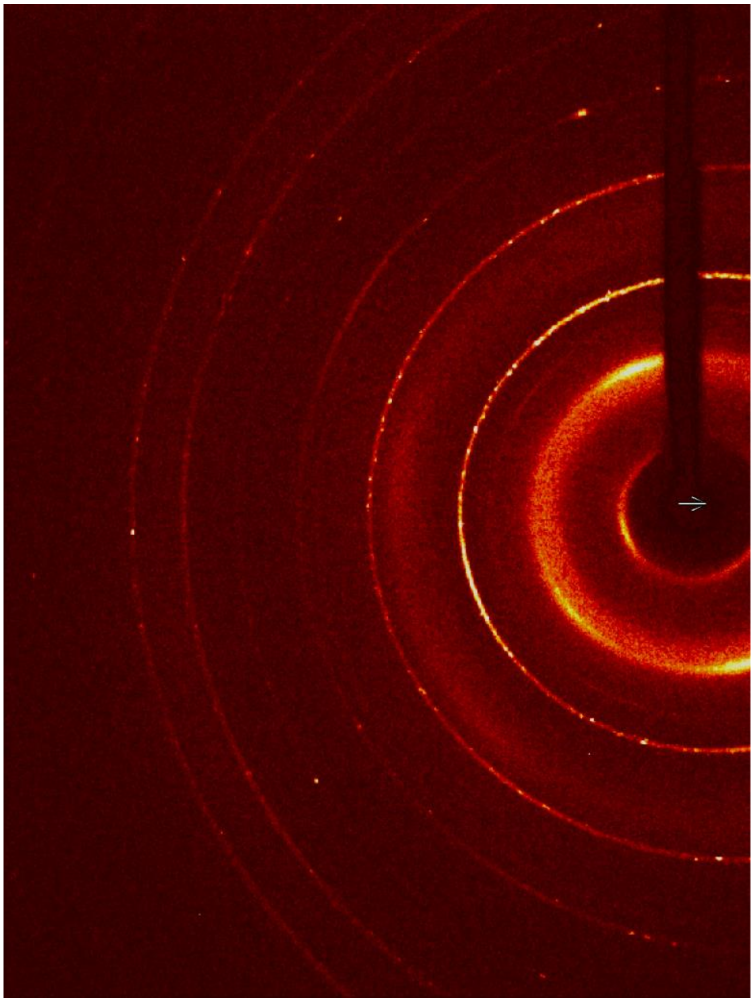

Figure S39. 2D WAXS image of PH3OHCOE-59 re-run.

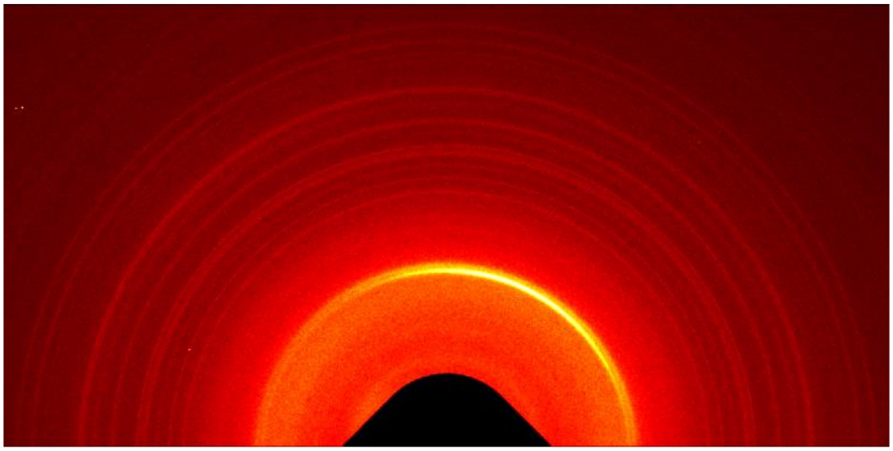

Figure S40. 2D WAXS image of PH(*R*)3OHCOE-58.

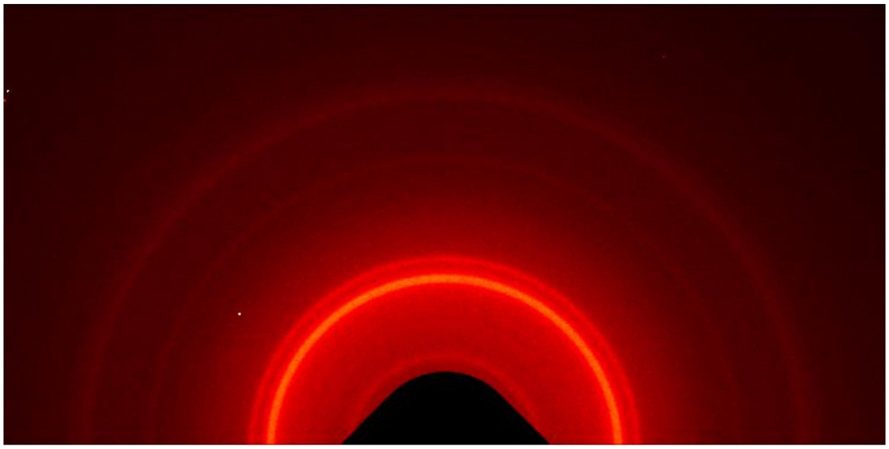

Figure S41. 2D WAXS image of EVOH(44)-26.

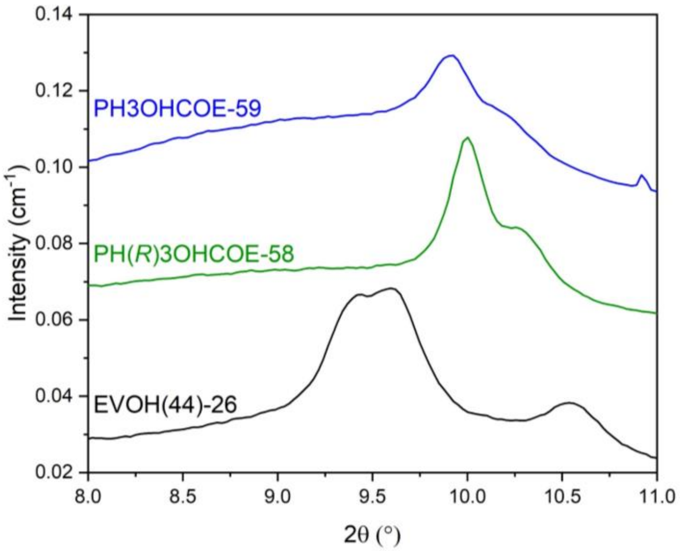

Figure S42. Overlay of 1D WAXS spectra for PH3OHCOE-59 (top), PH(*R*)3OHCOE-58 (middle), EVOH(44)-26 (bottom) from 8.00-11.00 ° (q = 1.20 Å^{-1}-1.65 Å^{-1}, λ = 0.729 Å).

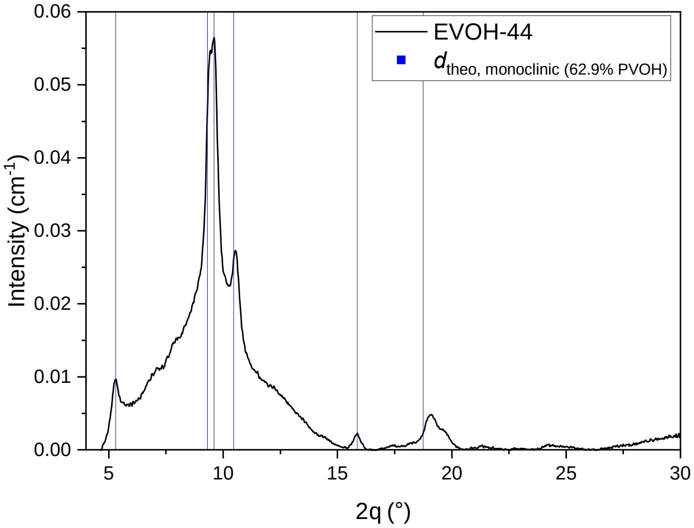

Figure S43. EVOH(44)-26 1D WAXS data overlayed with theoretical 2θ values. Literature *d* values were taken from the 62.9% sample (Matsumoto, T. *et al.*[6]) and converted to 2θ values via Bragg's law. The [100] peak was set to be at the maximum of the peak with the lowest 2θ value, and remaining theoretical *d* values were shifted accordingly.

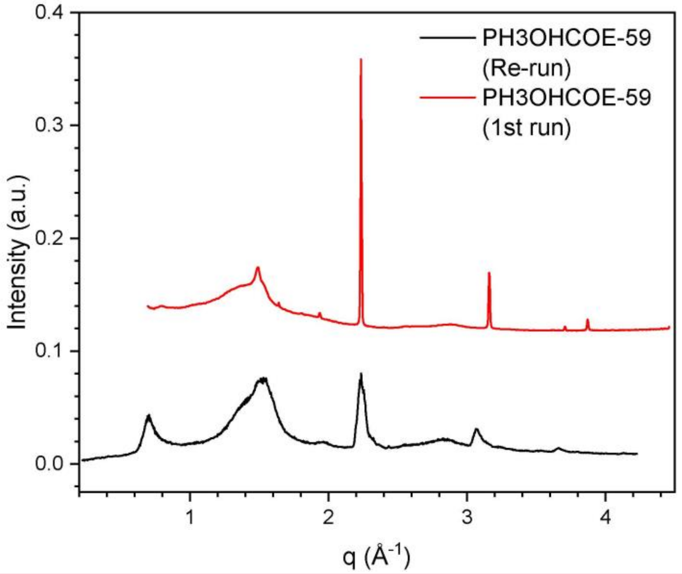

Figure S44. 1D WAXS data from original data collection (top) and sample re-run (bottom) of PH3OHCOE-59 to determine if intense peaks around 2.2 $Å^{-1}$ and 3.1 $Å^{-1}$ were from impurities or artifacts.

Commented [MH1]: Please expand caption to describe what you did in the re-run. Filter? Be explicit here.

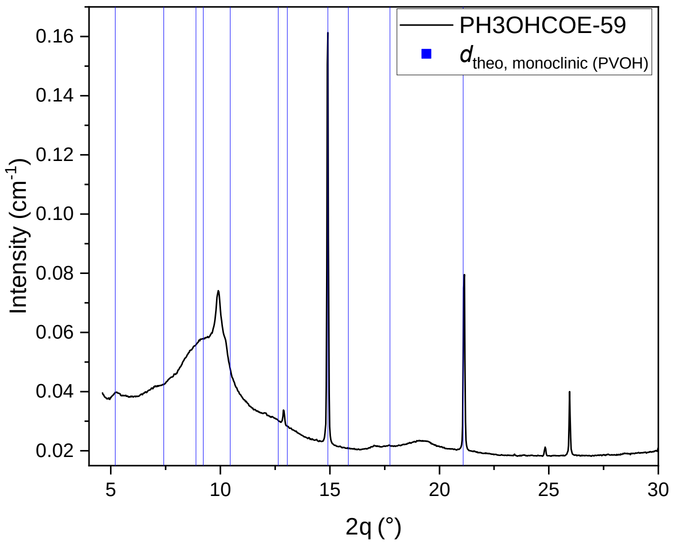

Figure S45. PH3OHCOE-59 1D WAXS data overlayed with theoretical 2θ values. Literature *d* values were taken from the PVOH sample (Matsumoto, T. *et al.*[6]) and converted to 2θ values via Bragg's law. Data shifting was not necessary for peak alignment of the initial [100] peak.

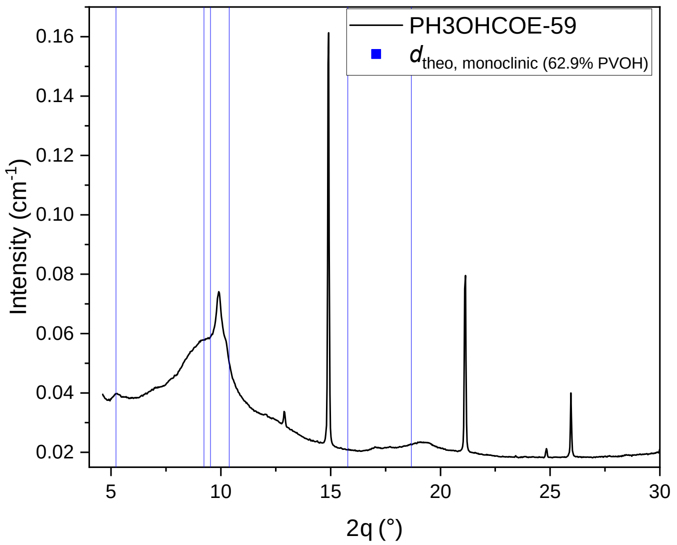

Figure S46. PH3OHCOE-59 1D WAXS data overlayed with theoretical 2θ values. Literature *d* values were taken from the 62.9% sample (Matsumoto, T. *et al.*[6]) and converted to 2θ values via Bragg's law. The [100] peak was set to be at the maximum of the peak with the lowest 2θ value, and remaining theoretical *d* values were shifted accordingly.

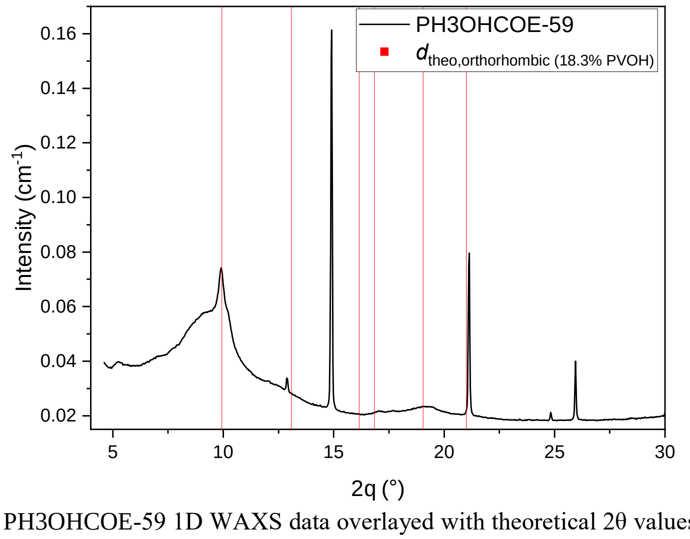

Figure S47. PH3OHCOE-59 1D WAXS data overlayed with theoretical 2θ values. Literature *d* values were taken from the 18.3% sample (Matsumoto, T. *et al.*[6]) and converted to 2θ values via Bragg's law. The [110] peak was set to be at the maximum of the most intense peak ~10 °, and remaining theoretical *d* values were shifted accordingly.

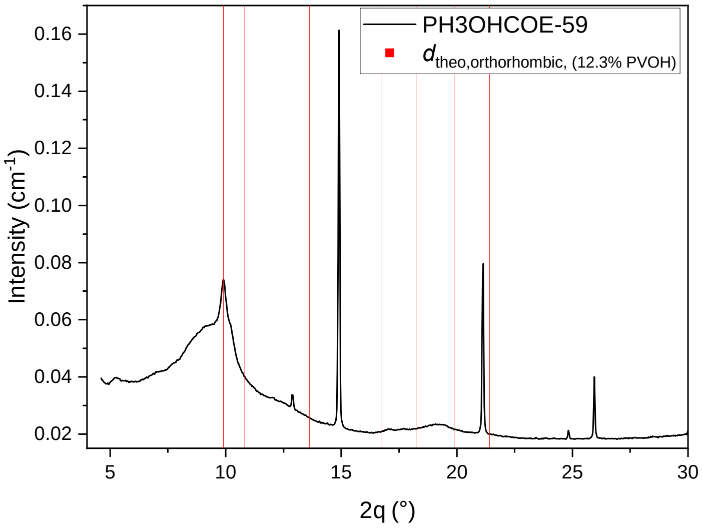

Figure S48. PH3OHCOE-59 1D WAXS data overlayed with theoretical 2θ values. Literature *d* values were taken from the 12.3% sample (Matsumoto, T. *et al.*[6]) and converted to 2θ values via Bragg's law. The [110] peak was set to be at the maximum of the most intense peak ~ 10 °, and remaining theoretical *d* values were shifted accordingly.

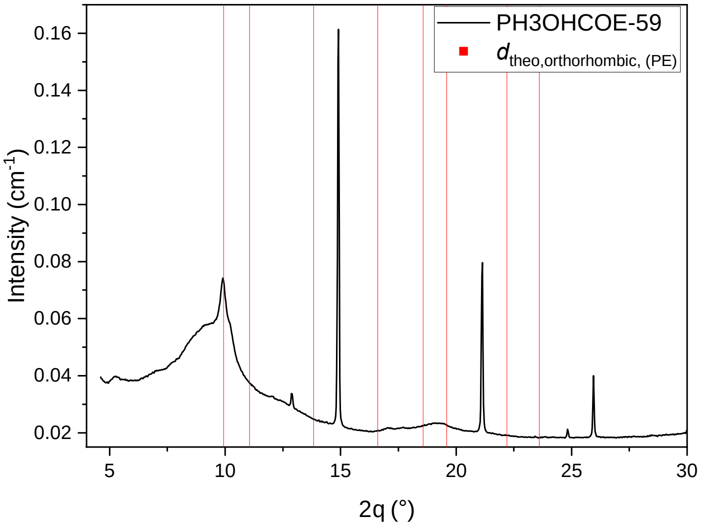

Figure S49. PH3OHCOE-59 1D WAXS data overlayed with theoretical 2θ values. Literature *d* values were taken from the PE sample (Matsumoto, T. *et al.*[6]) and converted to 2θ values via Bragg's law. Data shifting was not necessary for peak alignment of the initial [110] peak.

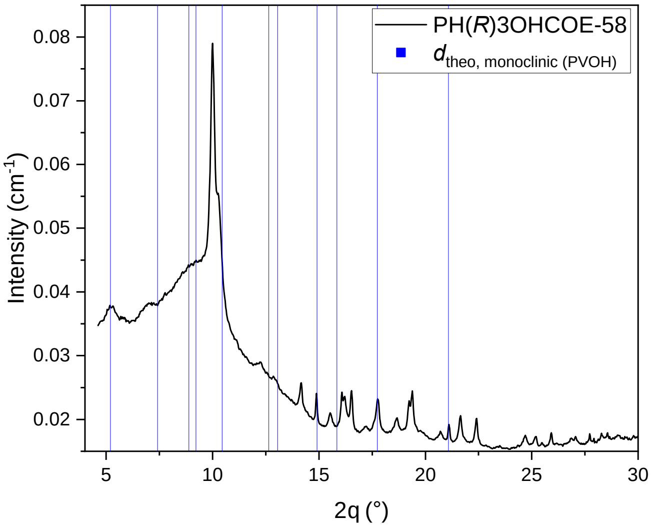

Figure S50. PH(*R*)3OHCOE-58 1D WAXS data overlayed with theoretical 2θ values. Literature *d* values were taken from the PVA sample (Matsumoto, T. *et al.*[6]) and converted to 2θ values via Bragg's law. Data shifting was not necessary for peak alignment of the initial [100] peak.

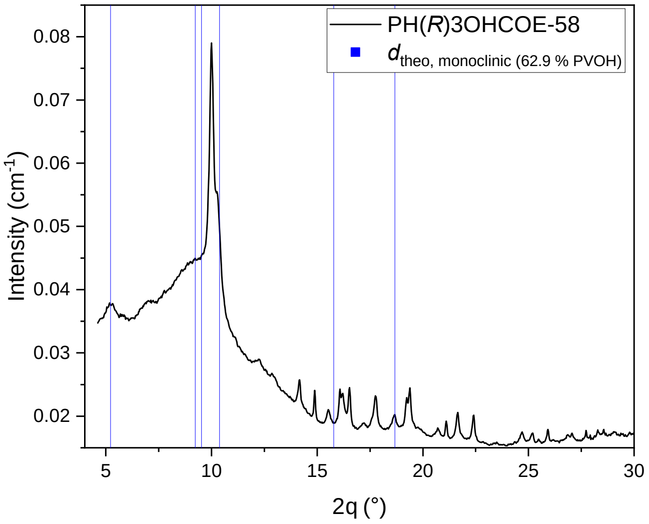

Figure S51. PH(*R*)3OHCOE-58 1D WAXS data overlayed with theoretical 2θ values. Literature *d* values were taken from the 62.9% sample (Matsumoto, T. *et al.*[6]) and converted to 2θ values via Bragg's law. The [100] peak was set to be at the maximum of the peak with the lowest 2θ value, and remaining theoretical *d* values were shifted accordingly.

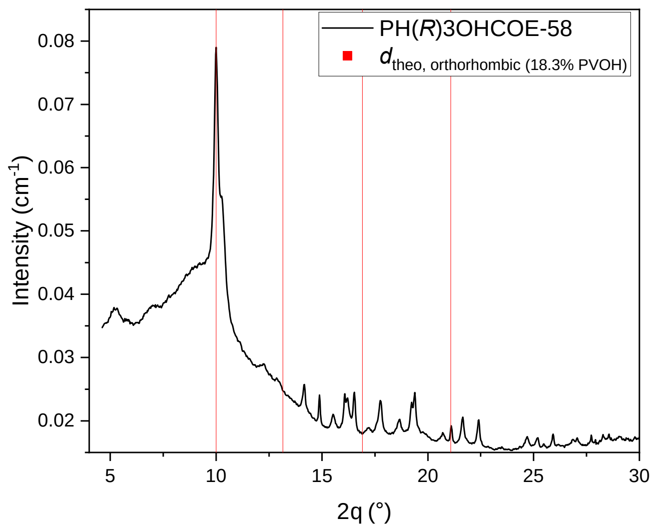

Figure S52. PH(R)3OHCOE-59 1D WAXS data overlayed with theoretical 2θ values. Literature d values were taken from the 18.3% sample (Matsumoto, T. *et al.*[6]) and converted to 2θ values via Bragg's law. The [110] peak was set to be at the maximum of the most intense peak, and remaining theoretical d values were shifted accordingly.

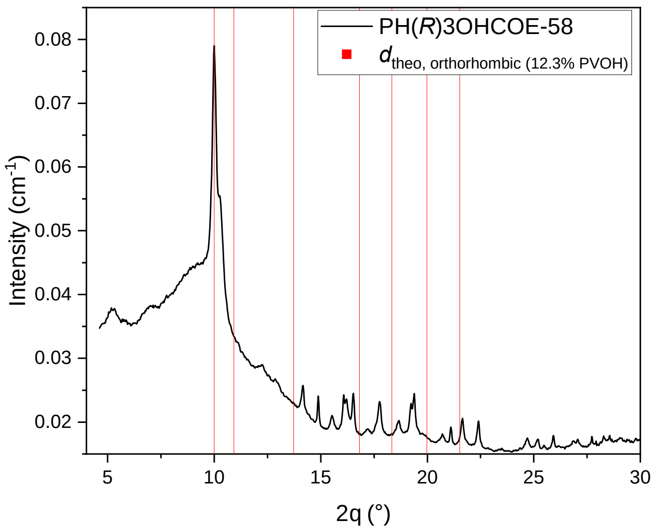

Figure S53. PH(R)3OHCOE-58 1D WAXS data overlayed with theoretical 2θ values. Literature d values were taken from the 12.3% sample (Matsumoto, T. *et al.*[6]) and converted to 2θ values via Bragg's law. The [110] peak was set to be at the maximum of the most intense peak, and remaining theoretical d values were shifted accordingly.

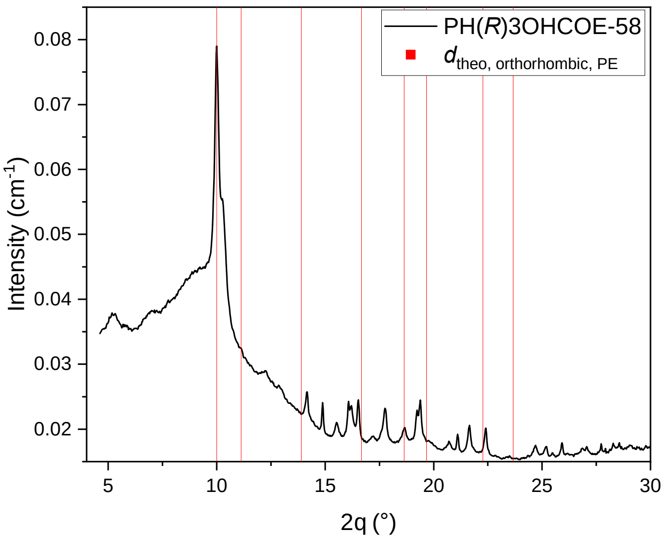

Figure S54. PH(*R*)3OHCOE-58 1D WAXS data overlayed with theoretical 2θ values. Literature *d* values were taken from the PE sample (Matsumoto, T. *et al.*[6]) and converted to 2θ values via Bragg's law. The [110] peak was set to be at the maximum of the most intense peak, and remaining theoretical *d* values were shifted accordingly.

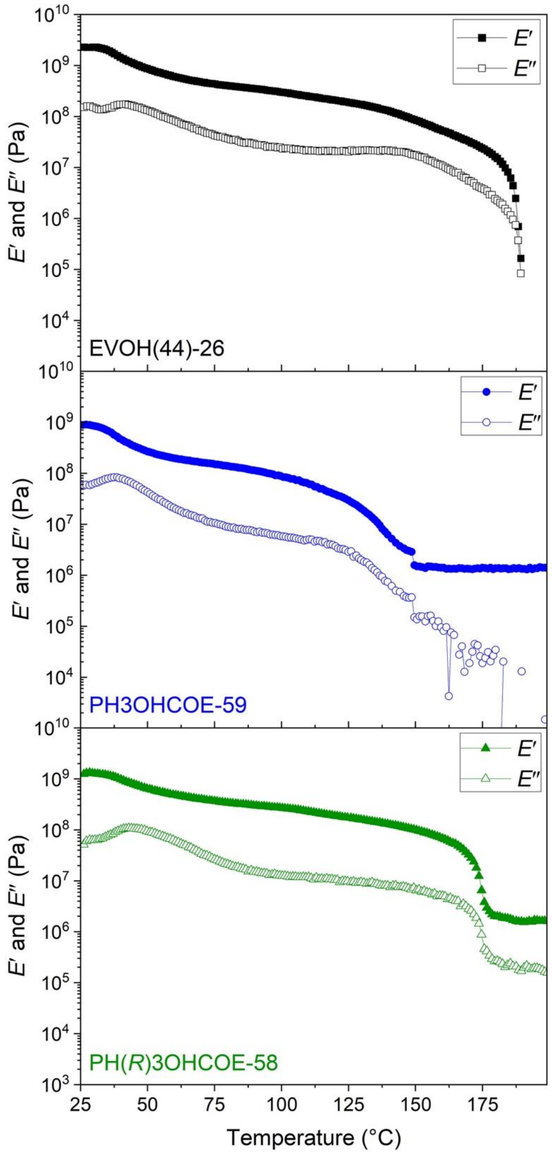

Figure S55. Extensional DMTA (5 °C·min^{-1}, 0.1 % strain, 1.0 Hz, 20.0g axial force) of EVOH(44)-26 (black square, top), PH3OHCOE-59 (blue circle, middle), and PH(*R*)3OHCOE-58 (green triangle, bottom). Filled symbols represent storage modulus (*E*′), and open symbols represent loss modulus (*E*″).

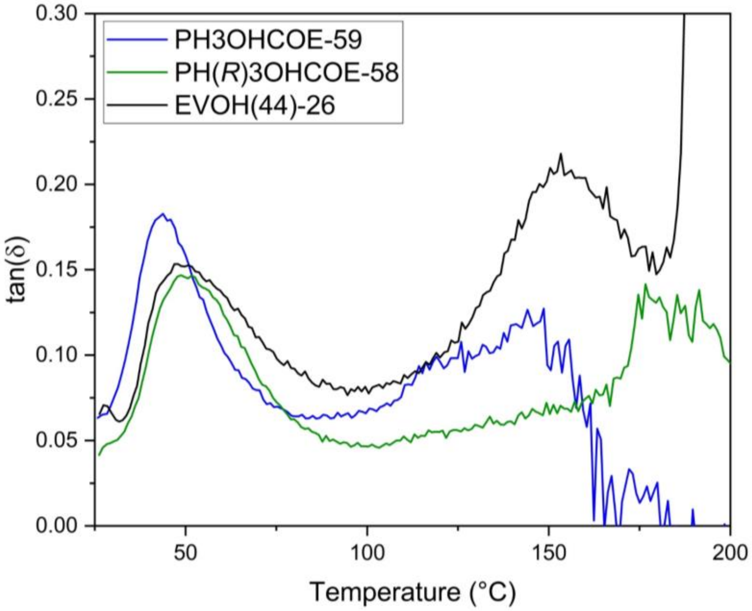

Figure S56. Extensional DMTA (5 °C·min^{-1}, 0.1 % strain, 1.0 Hz, 20.0g axial force) of EVOH(44)-26 (black), PH3OHCOE-59 (blue), PH(*R*)3OHCOE-58 (green) showing tan(δ) values.

Table S3. Comparison of T_g between DSC and DMTA.

	$T_{g, DSC}$ (°C)^a	$T_{g, DMTA}$ (°C)^b
PH3OHCOE-59	88	44
PH(*R*)3OHCOE-58	106	49
EVOH(44)-22	97	48

^{a}Determined by DSC from the first heating cycle (10 °C·min^{-1}). ^{b}Determined from DMTA by taking the temperature at the maximum value of tan(δ) in the range from 25-100 °C.

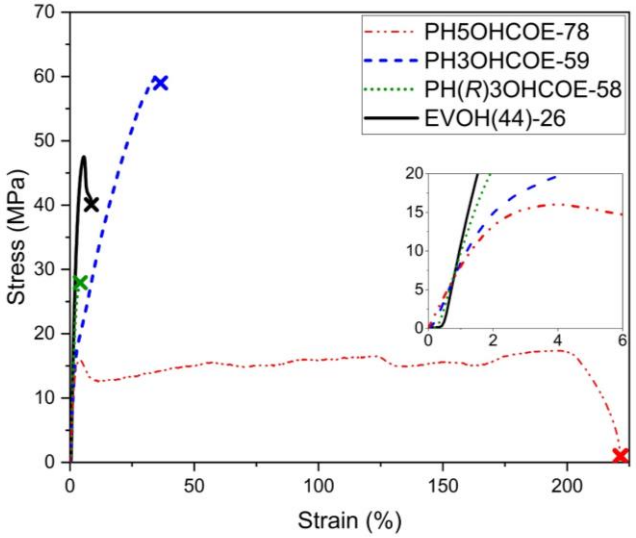

Figure S57. Uniaxial tensile analysis (1 mm·min^{-1}) of PH5OHCOE-78 (red dash-dot-dot), PH3OHCOE-59 (blue dash), PH(*R*)3OHCOE-58 (green dot), and EVOH(44)-26 (black line). X represents point of break. Inset shows *E* more clearly for each sample type.

Table S4. Oxygen barrier data for ROMP-derived polymers and EVOH(44)-26.

Commented [MH2]: Please double check I got this right.

Commented [DC(3R2]: Corrected (all very similar, just rounding discrepencies from using full non rounded numbers)

	Oxygen Transmission Rate [cc·(100 in^2·day)$^{-1}$]^a	Oxygen Permeability [cc·mil·(100 in^2·day·atm)$^{-1}$]^c	Thickness (mils)
PH5OHCOE-78	13.231, 10.473	31, 26 (avg = 29)	5.0
PH3OHCOE-59	0.687, 0.411	1.4, 0.9 (avg = 1.2)	4.5
PH3(*R*)OHCOE-58	1.050, 6.689	2.0, 11 (avg = 6.4)	3.6
EVOH(44)-26	0.303, 0.149	0.90, 0.51 (avg = 0.71)	6.5
	Water Transmission Rate [cc·(100 in^2·day)$^{-1}$]^b	Water Permeability [cc·mil·(100 in^2·day·atm)$^{-1}$]	Thickness (mils)
PH5OHCOE-78	0.260, 0.458	0.52, 0.86 (avg = 0.69)	4.7
PH3OHCOE-59	0.814, 0.494	1.8, 1.1 (avg = 1.5)	5.3
PH3(*R*)OHCOE-58	0.447, 0.714	1.0, 1.5 (avg = 1.2)	5.6
EVOH(44)-26	1.342, 2.066	3.4, 6.0 (avg = 4.7)	6.5

^{a}Measurements were taken at 0% humidity, 23 °C, 30 psi O_2 (2.0 atm). ^{b}Measurements were taken at 90% humidity, 38 °C, 35 psi (2.4 atm) H_2O. ^{c}At least two measurements were performed per sample type to confirm reproducibility, where both values are shown in oxygen transmission rate column. Data is presented as an average in oxygen permeability column.

References

(1) Zhang, J.; Matta, M. E.; Martinez, H.; Hillmyer, M. A. Precision Vinyl Acetate/Ethylene (VAE) Copolymers by ROMP of Acetoxy-Substituted Cyclic Alkenes. *Macromolecules* **2013**, *46* (7), 2535–2543.

(2) Dingwell, C. E.; Hillmyer, M. A. Regiospecific Poly(Ethylene-Co-Vinyl Alcohol) by ROMP of 3-Acetoxycyclooctene and Postpolymerization Modification for Barrier Material Applications. *ACS Appl. Polym. Mater.* **2023**, *5* (3), 1828–1836.

(3) Bartoli, G.; Bosco, M.; Carlone, A.; Dalpozzo, R.; Locatelli, M.; Melchiorre, P.; Palazzi, P.; Sambri, L. The First Simple Method of Protection of Hydroxy Compounds as Their O-Boc Derivatives under Lewis Acid Catalysis. *Synlett* **2006**, *2006* (13), 2104–2108.

(4) Gais, H.-J.; Jagusch, T.; Spalthoff, N.; Gerhards, F.; Frank, M.; Raabe, G. Highly Selective Palladium Catalyzed Kinetic Resolution and Enantioselective Substitution of Racemic Allylic Carbonates with Sulfur Nucleophiles: Asymmetric Synthesis of Allylic Sulfides, Allylic Sulfones, and Allylic Alcohols. *Chemistry – A European Journal* **2003**, *9* (17), 4202–4221.

(5) Hoye, T. R.; Jeffrey, C. S.; Shao, F. Mosher Ester Analysis for the Determination of Absolute Configuration of Stereogenic (Chiral) Carbinol Carbons. *Nat. Protoc.* **2007**, *2* (10), 2451–2458.

(6) Matsumoto, T.; Nakamae, K.; Ogoshi, N.; Kawasoe, M.; Oka, H. The Crystallinity of Ethylene-Vinyl Alcohol Copolymers. *Kobunshi Kagaku* **1971**, *28* (315), 610–617.
